# MDM2 inhibitor APG-115 synergizes with PD-1 blockade through enhancing antitumor immunity in the tumor microenvironment

**DOI:** 10.1186/s40425-019-0750-6

**Published:** 2019-11-28

**Authors:** Douglas D. Fang, Qiuqiong Tang, Yanhui Kong, Qixin Wang, Jiaxing Gu, Xu Fang, Peng Zou, Tao Rong, Jingwen Wang, Dajun Yang, Yifan Zhai

**Affiliations:** 1 Ascentage Pharma (Suzhou) Co, Ltd, 218 Xinghu Street, Suzhou, Jiangsu Province China; 2 Oncology & Immunology Unit, WuXi Apptec (Suzhou) Co, Ltd, 1318 Wuzhong Avenue, Suzhou, Jiangsu Province China; 3 Department of Experimental Research, State Key Laboratory of Oncology in South China, Collaborative Innovation Center for Cancer Medicine, Sun Yat-Sen University Cancer Center, Guangzhou, China

**Keywords:** MDM2 inhibitor, p53, APG-115, Macrophage, Anti-PD-1, Immuno-oncology, Tumor microenvironment

## Abstract

**Background:**

Programmed death-1 (PD-1) immune checkpoint blockade has achieved clinical successes in cancer therapy. However, the response rate of anti-PD-1 agents remains low. Additionally, a subpopulation of patients developed hyperprogressive disease upon PD-1 blockade therapy. Combination therapy with targeted agents may improve immunotherapy. Recent studies show that p53 activation in the myeloid linage suppresses alternative (M2) macrophage polarization, and attenuates tumor development and invasion, leading to the hypothesis that p53 activation may augment antitumor immunity elicited by anti-PD-1 therapy.

**Method:**

Using APG-115 that is a MDM2 antagonist in clinical development as a pharmacological p53 activator, we investigated the role of p53 in immune modulation and combination therapy with PD-1 blockade.

**Results:**

In vitro treatment of bone marrow-derived macrophages with APG-115 resulted in activation of p53 and p21, and a decrease in immunosuppressive M2 macrophage population through downregulation of c-Myc and c-Maf. Increased proinflammatory M1 macrophage polarization was observed in the spleen from mice treated with APG-115. Additionally, APG-115 has co-stimulatory activity in T cells and increases PD-L1 expression in tumor cells. In vivo, APG-115 plus anti-PD-1 combination therapy resulted in enhanced antitumor activity in *Trp53*^*wt*^, *Trp53*^*mut*^, and *Trp53-*deficient (*Trp53*^*−/−*^) syngeneic tumor models. Importantly, such enhanced activity was abolished in a syngeneic tumor model established in *Trp53* knockout mice. Despite differential changes in tumor-infiltrating leukocytes (TILs), including the increases in infiltrated cytotoxic CD8^+^ T cells in *Trp53*^*wt*^ tumors and M1 macrophages in *Trp53*^*mut*^ tumors, a decrease in the proportion of M2 macrophages consistently occurred in both *Trp53*^*wt*^ and *Trp53*^*mut*^ tumors upon combination treatment.

**Conclusion:**

Our results demonstrate that p53 activation mediated by APG-115 promotes antitumor immunity in the tumor microenvironment (TME) regardless of the *Trp53* status of tumors per se. Instead, such an effect depends on p53 activation in *Trp53* wild-type immune cells in the TME. Based on the data, a phase 1b clinical trial has been launched for the evaluation of APG-115 in combination with pembrolizumab in solid tumor patients including those with *TP53*^*mut*^ tumors.

## Introduction

The successful development of immune checkpoint inhibitors, such as monoclonal antibodies against programmed cell death 1 (PD-1) and PD-1 ligand (PD-L1), is revolutionizing cancer therapy. While some patients treated with anti-PD-(L)1 agents have experienced dramatic tumor regressions, a significant subset of patients failed to respond to anti-PD-(L)1 immunotherapy. Moreover, 9–29% patients may develop a hyperprogressive disease [1–3]. The exact mechanisms associated with hyperprogressive disease remain largely unknown. MDM2 amplification identified in some of these patients indicates that the genetic alteration may contribute to hyperprogressive disease [3], and raises the possibility that a combination strategy with MDM2 inhibitors could limit hyperprogression on immunotherapy. In addition, recent studies suggested a crucial role of macrophage reprogramming, upon Fc receptor engagement by immune checkpoint inhibitor, in the development of hyperprogressive disease in non-small cell lung cancer patients [4].

Pre-existing tumor microenvironment (TME) also influences the responsiveness to immunotherapy [5, 6]. Positive prognostic factors for responses to anti-PD-(L)1 immunotherapy includes PD-L1 expression, high tumor mutational burden, infiltration of TILs, expression of neoantigens, expression of PD-L1 on dendritic cells and macrophages, and an IFN-γ gene signature [7–14]. Conversely, tumors that are devoid of T cells or enriched with immunosuppressive immune cells, such as tumor-associated macrophages (TAMs) and myeloid-derived suppressor cells (MDSCs), are less likely to respond to PD-1/PD-L1 blockade [15].

Therapies that can create or promote an antitumor microenvironment that is otherwise immune suppressed or immunologically barren have the potential to improve the therapeutic response to anti-PD-(L)1 immunotherapy. TAMs are a heterogeneous population of myeloid cells present in the TME, which can be further defined as proinflammatory (i.e., antitumoral) M1 and immunosuppressive (i.e., protumoral) M2 macrophages according to their phenotypic and functional states [16, 17]. M1 macrophages associated with a proinflammatory cytokine response are involved in efficient antigen presentation and promote T helper type 1 cell response, all of which inhibit tumor progression. Conversely, M2 macrophages are associated with immunosuppression that enables the establishment and development of tumors as well as metastatic dissemination [18, 19]. To achieve self-sustainability, cancer cells create a microenvironment that is enriched in signals that skew TAMs towards an M2-like lineage. Such a TME suppresses antitumor immune response and, in turn, promotes tumor progression and metastasis [20–22]. Therefore, depletion of TAMs, or changing the M2/M1 ratio towards the M1 lineage, has emerged as an attractive therapeutic approach [23–25].

A recent study using genetic methods demonstrated that p53 restoration or re-activation in myeloid cells led to tumor regression and clearance, which was at least partially caused by the activation of innate antitumor immunity [26, 27]. Furthermore, a role for p53 in M1 and M2 macrophage polarization has been suggested [28–30]. Mild p53 activation in the myeloid lineage attenuated tumor development and invasion, and suppressed alternative (M2) macrophage polarization together with c-Myc downregulation [30]. Collectively, p53 activation in the macrophages may act as a regulator of their functions and consequently suppress tumorigenesis by promoting an antitumor microenvironment.

The activity of p53 is primarily controlled by the ubiquitin E3 ligase mouse double minute 2 homolog (MDM2), which maintains low intracellular levels of p53 by targeting it for proteasomal degradation and inhibiting its transcriptional activity. As an oncogene that commonly is over-expressed in human cancers, MDM2 represents a novel target for cancer therapy. Several MDM2 antagonists have been developed to disrupt the MDM2-P53 protein-protein interaction in order to restore the normal active conformation of P53 in *TP53* wild-type (*TP53*^*wt*^) tumors. APG-115 is an orally active, selective, potent small molecule inhibitor of the MDM2-P53 protein-protein interaction, which destabilizes MDM2-P53 complex and promotes P53 activation [31]. APG-115 as a single agent or in combination with Pembrolizumab is currently in clinical trials in patients with solid tumors (ClinicalTrials.gov identifier NCT02935907 and NCT03611868).

In this study, using murine cells and tumor models, we asked whether targeting MDM2-p53 pathway by APG-115 regulated immune responses and augmented antitumor immunity elicited by anti-PD-1 therapy. Our results demonstrate that p53 activation in the immune cells in the TME by APG-115 treatment promotes antitumor immunity. APG-115 enhances antitumor efficacy of anti-PD-1 antibody in *Trp53*^*wt*^, *Trp53*^*mut*^, and *Trp53-*deficient (*Trp53*^*−/−*^) syngeneic tumor models. Mechanistically, besides increased infiltration of cytotoxic CD8^+^ T cells and M1 macrophages in the TME of *Trp53*^*wt*^ tumors, decreased infiltration of M2 macrophages also contributes to the conversion of immunosuppressive to immunostimulatory TME in both *Trp53*^*wt*^ and *Trp53*^*mut*^ settings. Interestingly, in *Trp53*-knockout mice where the endogenous *Trp53* gene is completed deleted, APG-115 treatment failed to enhance anti-PD-1 efficacy, implicating for the requirement of intact p53 in order to activate p53 protein in the immune cells in the host animals. Taken together, our study suggests that promoting an antitumor microenvironment with a MDM2 antagonist such as APG-115 may enhance efficacy of PD-1 blockade in clinic and, importantly, such an effect is independent of the p53 status of tumors per se.

## Materials and methods

### Cell lines and reagents

Anti-PD-1 (clone RMP1–14) and rat IgG2a isotype control antibody (clone 2A3) were purchased from BioXcell. APG-115 (Ascentage Pharma) was dissolved in DMSO (Sigma) to make a stock solution for in vitro use. MC38 cell line derived from a C57BL/6 murine colon adenocarcinoma and MH-22A cell line derived from C3H murine liver cancer were obtained from Sun Yat-Sen University Cancer Center (Guangzhou, China) and European Collection of Authenticated Cell Cultures, respectively. All cell lines were genetically authenticated and free of microbial contamination.

### In vivo experiments

Six- to eight-week old female mice were obtained from Beijing Vital River Laboratory Animal Technology Co., Ltd. (Beijing, China). Mice were implanted subcutaneously with MC38 (0.5 × 10^6^, C57BL/6), MH-22A (5 × 10^6^, C3H), or *Trp53*^*−/−*^ MH-22A (5 × 10^6^, C3H) cells in 0.1 mL PBS per animal to establish syngeneic tumor models. When the average tumor size reached 50–100 mm^3^, tumor-bearing mice were randomly assigned into groups based on their tumor volumes. *Trp53*^*−/−*^ knockout C57BL/6 J mice were purchased from Biocytogen (Beijing, China).

APG-115 was formulated in a vehicle of 0.2% HPMC (Sigma Aldrich) and administered orally at 10 or 50 mg/kg daily or every other day (Q2D). Anti-PD-1 antibody was diluted in PBS and dosed intraperitoneally at 5 or 10 mg/kg twice a week (BIW). Vehicle plus isotype control antibody, or vehicle only were used as the control. Tumor volume (V) was expressed in mm^3^ using the following formula: V = 0.5 a × b^2^; where a and b were the long and short diameters of the tumor, respectively. As a measurement of efficacy, a T/C (%) value was calculated at a time point according to: T/C (%) = (T_RTV_/C_RTV_) × 100%; where T_RTV_ was the relative tumor volume (RTV) of the treatment group and C_RTV_ was the RTV of the control group. RTV = V_t_/V_1_; where V_1_ and V_t_ were the average tumor volumes on the first day of treatment (day 1) and the average tumor volumes on a certain time point (day t), respectively. Additional measurements of response included stable disease (SD), partial tumor regression (PR), and complete regression (CR) were determined by comparing tumor volume change at day t to its baseline: tumor volume change (%) = (V_t_-V_1_/V_1_). The BestResponse was the minimum value of tumor volume change (%) for t ≥ 10. For each time point t, the average of tumor volume change from t = 1 to t was also calculated. BestAvgResponse was defined as the minimum value of this average for t ≥ 10. The criteria for response (mRECIST) were adapted from RECIST criteria [32, 33] and defined as follows: mCR, BestResponse < − 95% and BestAvgResponse < − 40%; mPR, BestResponse < − 50% and BestAvgResponse < − 20%; mSD, BestResponse < 35% and BestAvgResponse < 30%; mPD, not otherwise categorized. SD, PR, and CR were considered responders and used to calculate response rate (%). Body weight of animals were monitored simultaneously. The change in body weight was calculated based on the animal weight of the first day of dosing (day 1). Tumor volume and changes in body weight (%) were represented as the mean ± standard error of the mean (SEM).

In re-challenge studies, naïve mice and CR mice were inoculated subcutaneously with 5 × 10^6^ MH-22A tumor cells per animal. Tumor growth was monitored for 3 weeks without further treatment.

Animal studies were conducted in the animal facility of GenePharma (Suzhou, China). The protocols and experimental procedures involving the care and use of animals were approved by the GenePharma Institutional Animal Care and Use Committee.

### Flow cytometry

For analysis of TILs in the TME, isolated tumors were weighed and dissociated by gentle MACS buffer (Miltenyi) and then filtered through 70 μm cell strainers to generate single-cell suspensions. After counting viable cells, the samples were incubated with a live-dead antibody followed by FcγIII/IIR-blocking staining. Cells were then stained with fluorochrome-labeled antibodies against CD45 (Thermo Fisher Scientific, catalog #69–0451-82), CD4 (BD Biosciences, catalog #552775), CD8 (Thermo Fisher, catalog #45–0081-82), CD3 (Thermo Fisher, catalog #11–0032-82), CD49b (Thermo Fisher, catalog #48–5971-82), CD11b (Thermo Fisher, catalog #48–0112-82), F4/80 (Thermo Fisher, catalog #17–4801-82), CD206 (Thermo Fisher, catalog #12–2061-82), MHC-II (Thermo Fisher, catalog #11–5321-82), Gr-1 (Biolegend, catalog #108439), CD25 (BD, catalog #564370), NK1.1 (eBioscience, catalog #48–5941-82), and Foxp3 (Thermo Fisher, catalog #12–5773-82).

For analysis of cytokines in TILs, single cell suspensions generated from isolated tumors were plated into six-well plates and stimulated with PMA (50 ng/mL) and ionomycin (500 ng/mL) for 4 hours. Two hours before the end of stimulation, protein transport inhibitor monensin (2 μM) was added. Cells were collected and incubated with a live-dead antibody followed by FcγIII/IIR-blocking staining. Cells were then stained with fluorochrome-labeled antibodies against CD45, CD4, CD8, CD3, IFN-γ (BD, catalog #554413) and TNF-α (BD, catalog #554419).

For PD-L1 expression, MH-22A cells were treated with indicated concentrations of APG-115 for 72 h. PD-L1 (BD, catlog #563369) expression and its fluorescence intensity were acquired on an Attune NxT flow cytometer (Life Technology) and analyzed using Flowjo software (BD).

### Generation and analysis of bone marrow-derived macrophages (BMDMs)

Bone marrow cells were collected from two femurs of each mouse and plated in complete RPMI-1640 medium supplemented with 10% FBS, 100 ng/mL m-CSF (R&D, catlog # 416-ML-050), and 1% penicillin and streptomycin (Invitrogen). After 7 days, cells were harvested and evaluated by flow cytometry for expression of CD11b and F4/80. BMDMs were further treated with IL-4 (20 ng/mL, R&D) to induce alternative (M2) macrophage polarization with or without APG-115 (250 nM or 1 μM). Cells were then harvested and evaluated for the expression of M2 markers (MHC-II and CD206) by flow cytometry, expression of M2-related genes (*Arg-1* and *Retnla*) by RT-qPCR, and p53, p21, c-Myc and c-Maf protein levels by Western blotting.

### RT-qPCR analysis

After treatment, BMDMs were harvested and mRNA was extracted using a RNAEasy mini plus kit (Qiagen). cDNA was retro-transcribed from 1 μg of RNA primed with random hexamers using cDNA reverse transcription kit (Takara), 3 ng of equivalent cDNA were amplified in a qPCR (SyBr) assay on an ABI7500 (Thermo Fisher) for the following genes: Arg-1 (SyBr green PCR using primers 5′- CATTGGCTTGCGAGACGTAGAC and 5′- GCTGAAGGTCTCTTCCATCACC) and Retnla (SyBr green PCR using primers 5′- CAAGGAACTTCTTGCCAATCCAG and 5′- CCAAGATCCACAGGCAAAGCCA). Relative gene expression was quantified with the 2-delta method, normalized to GAPDH housekeeping gene detected by SyBr green RT-PCR (5′-AACTTTGGCATTGTGGAAGG and 5′- GGATGCAGGGATGATGTTCT).

### Western blotting

Cells were collected and lysed in RIPA lysis buffer (Yeasen, catalog #20101ES60) containing a protease inhibitor cocktail (Yeasen, catalog #20104ES08). Protein concentration was quantified by bicinchoninic acid assay (Thermal Fisher). Equal amounts of soluble protein were loaded and separated on a 10% SDS-PAGE, followed by transfer to nitrocellulose and then immunoblotting using primary antibodies, including p53 (CST, catalog #32532), p21 (abcam, catalog #ab109199), c-Myc (CST, catalog #13987 T), c-Maf (abcam, catalog #ab77071), p-STAT3 (CST, catalog #9145), t-STAT3 (CST, catalog #9139), PD-L1 (R&D, catalog #AF1019), Caspase 3 (CST, catalog #9665S), ZAP70 (CST, catalog #3165S), MDM2 (BD, catalog #556353), and β-actin (CST, catalog #3700S). HRP-conjugated secondary antibodies (Yeasen, catalog #33101ES60, catalog #33201ES60) were used at 1:5000 dilution.

### Analyses of T cell activation and proliferation

CD4^+^ T cells were positively selected from mouse spleens using magnetic beads (Miltenyi, catalog #130–049-201) and stimulated with 10 μg/mL plate-bound anti-CD3 (eBioscience, catalog #16–0031-85) and 2 μg/mL anti-CD28 (eBioscience, catalog #16–0281-85) in the presence of 250 nM APG-115 or DMSO for 1 or 2 days. After treatment, cells were harvested and evaluated by flow cytometry for the expression of CD25 (BD, catalog #557192), CD62L (BD, catalog #553151), and Foxp3 (Thermo Fisher, catalog #12–5773-82). T cell activation were defined as CD25^high^CD62L^low^ and enlarged cell size. CD25^+^Foxp3^+^T cells represented Treg population.

For T cell proliferation, CD4^+^ T and CD8^+^ T cells were positively selected from mouse spleens using magnetic beads (Miltenyi, catalog #130–049-201 and #130–096-495) and then stimulated with a series of concentrations of plate-bound anti-CD3 and 2 μg/mL anti-CD28 in the presence of 250 nM APG-115 or DMSO. After 72 h, relative cell numbers were determined using CellTiter-Glo luminescent cell viability assay (Promega, catalog #G7571) and normalized to unstimulated cultures treated with DMSO control.

### Cytotoxic T lymphocyte killing assay

OT-I splenocytes were stimulated with 2 μg/mL OVA peptide (SIINFEKL, GL Biochem, catalog #53698) and 10 ng/mL rmIL-2 (R&D, catalog #402-ML-500) for 72 h in the complete RPMI-1640 medium supplied with vehicle, 50 nM, 250 nM, or 1 μM APG-115. Cells were harvested after the treatment. EL4 cells (target cells, T) were labeled with 50 nM CellTrace Far-Red dye (Invitrogen, catalog #C34564) and then pulsed with 20 μg/mL OVA peptide for 30 min at 37 °C in the complete RPMI-1640 medium. Labeled EL4 cells (2 × 10^4^) were seeded in each well of a 96-well plate. OT-I CD8^+^ T cells (effector cells, E) treated with four different conditions were seeded with targeted EL4 cells in an E:T ratio of 0:0, 0.5:1, 2:1, or 8:1. The effector and target cells were co-cultured overnight. PI dye was added to the mixed cell solutions at 1:10000 and incubated for 10 min. Percentage of target cell lysis were analyzed using FACS LSRFortessa (BD).

### Statistical analyses

One-way ANOVA followed by Bonferroni’s posttest was applied to assess the statistical significance of differences between multiple treatment groups. All data were analyzed in SPSS version 18.0 (IBM, Armonk, NY, U.S.A.). Prism version 6 (GraphPad Software Inc., San Diego, CA, U.S.A.) was used for graphic presentation.

## Results

### APG-115 suppresses alternative (M2) macrophage polarization and increases M1 macrophage polarization

**Fig. 1:**
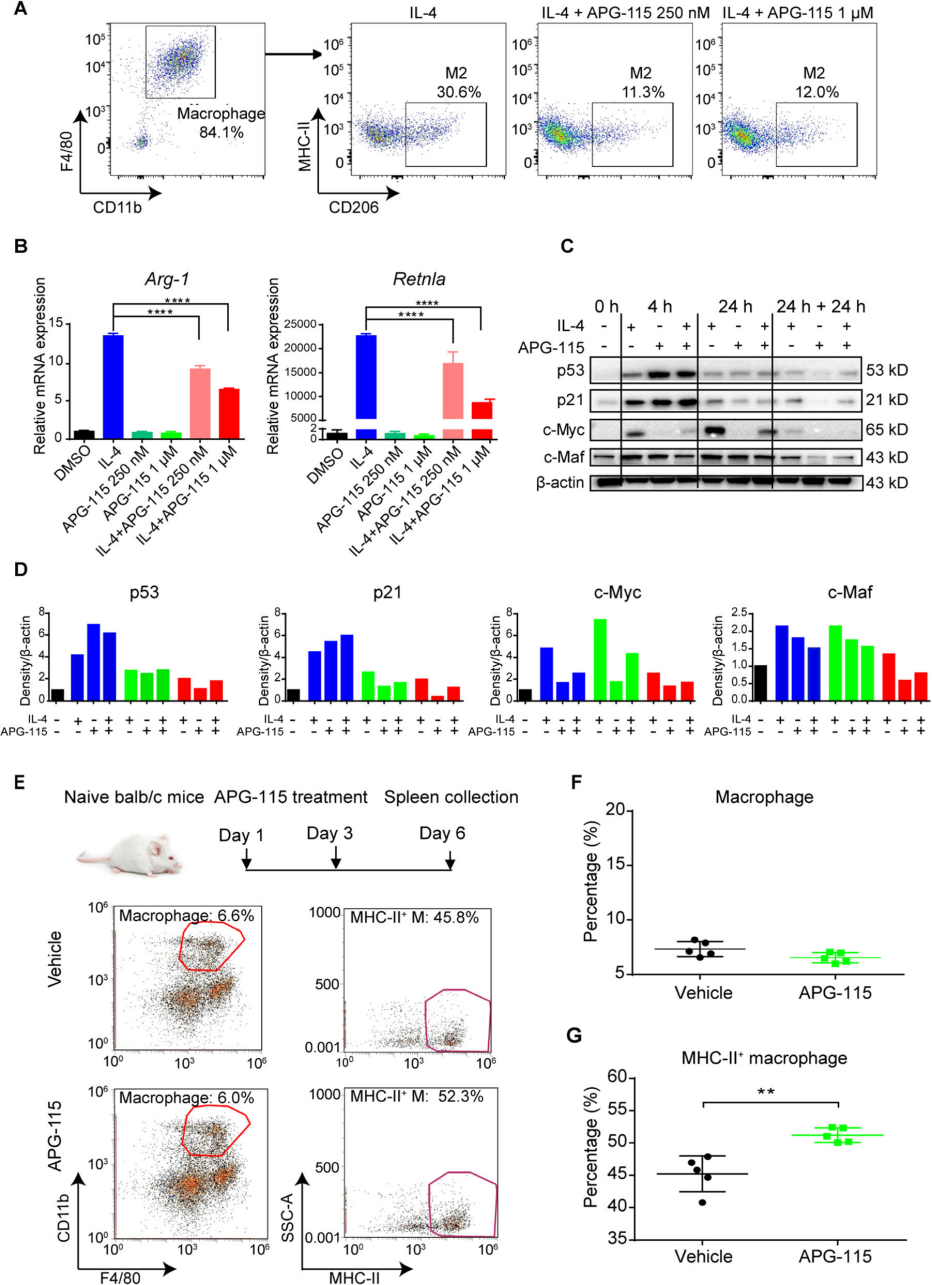
APG-115 suppresses alternative M2 macrophages polarization in vitro and increases M1 macrophages in vivo through activation of p53 pathway. **a** BMDMs were generated under the treatment with m-CSF for 7 days and then treated with IL-4 (20 ng/mL) to induce alternative macrophage polarization (M2) for 24 h in the absence or presence of APG-115. Cells were then harvested for detection of M2 macrophages (CD206^+^MHC-II^low^) by flow cytometry. **b** the mRNA expression levels of *Arg-1* and *Retnla* in the above BMDMs induced by the treatment with IL-4 (20 ng/mL) with or without APG-115 were analyzed by RT-qPCR. Duplicated samples were tested. **c** Western blot analysis of p53, p21, c-Myc and c-Maf total proteins in BMDMs treated with IL-4 (20 ng/mL) with or without APG-115 (1 μM) for 0, 4, or 24 h, or sequentially treated with IL-4 and then APG-115 for 24 h each (24 h + 24 h). **d** Quantification of C. 0 (black bars), 4 (blue bars), or 24 h (green bars), or sequentially treated with each agent for 24 h (24 h + 24 h, red bars). **e** naïve BALB/c mice were treated with APG-115 (10 mg/kg, Q2D × 2 doses; *n* = 5). Two days after the last dose, spleens were collected, dissociated into single-cell suspensions, and stained with macrophage markers for flow cytometry analysis. Macrophages were defined as CD11b^+^ F4/80^hi^, and further analyzed for M1 macrophages by expression of MHC-II. Pooled data of percentages of macrophages gated on CD45^+^CD3^−^ live cells (**f**) and percentages of M1 macrophages gated on macrophages (**g**) from five mice were plotted

Considering the essential role of p53 in M1 function and M2 polarization [28–30], we first explored how APG-115-mediated p53 activation affected M1 and M2 macrophages. Briefly, BMDMs were generated and confirmed by expression of CD11b^+^F4/80^hi^ using a flow cytometer (Fig. 1a, left panel). After stimulated under a M2-polarizing condition in the presence of 20 ng/mL IL-4 for 24 h, a substantial population (30.6%) of CD206^+^MHC-II^−^ M2 macrophages were induced (Fig. 1a, middle panel). Concurrent treatments with 250 nM or 1 μM APG-115 (IL-4 + APG-115) inhibited M2 polarization, resulting in only 11 and 12% M2 macrophages, respectively (Fig. 1a, right panels). RT-PCR analysis showed that mRNA expression of M2-associated genes (i.e., *Arg-1* and *Retnla*) were substantially upregulated after IL-4 treatment for 48 h (Fig. 1b). Under concurrent treatment with APG-115, IL-4-induced mRNA expression of M2 associated genes was significant suppressed. These results demonstrate that APG-115 suppresses M2 macrophage polarization in vitro.

Expression of p53 and its key transcriptional target p21 was examined in M2-polarized macrophages. Western blot analysis revealed that p53 and p21 total proteins were significantly increased when macrophages were polarized to M2 subtype under IL-4 treatment for 4 h. Both proteins were further elevated upon co-treatment with APG-115 for 4 hours and the effect faded when treatment lasted for 24 h (Fig. 1c-d). c-Myc is a key regulator in alternative (M2) macrophage activation and c-Myc blockade in macrophages hampers IL-4 dependent induction of M2-associated genes [34]. In addition, transcription factor c-Maf is highly expressed in mouse and human polarized M2 macrophages [35, 36]. Our results revealed that, although strong induction of c-Myc was observed after exposure to IL-4, significant downregulation of c-Myc and c-Maf were found in cells co-treated with IL-4 and APG-115. The suppressive effect of APG-115 on c-Myc and c-Maf sustained after treatment with APG-115 for 24 h, whereas the activating effect of APG-115 on p53 and p21 disappeared. Similar to concurrent treatment, expression of c-Myc and c-Maf was also significant downregulated in cells under sequential treatment with IL-4 and APG-115. These results indicate that APG-115 indeed activates p53 and p21 expression in a time-dependent manner in BMDMs and, furthermore, suppresses c-Myc and c-Maf, which are critical regulators for M2 macrophage polarization.

Next, to explore the effect of APG-115 on M1 macrophages, naïve BALB/c mice were administered with APG-115 (Fig. 1e). Two days after the last dose, mouse splenocytes were collected and stained with macrophage markers. Macrophages were defined as CD11b^+^F4/80^hi^ and further analyzed for MHC-II by flow cytometry. No significant changes in the proportion of total macrophages were observed in mice after APG-115 treatment; however, the frequency of M1 macrophages, defined as MHC-II^+^, was significantly increased (Fig. 1f-g). The results suggest that APG-115 induces M1 macrophage polarization in vivo.

Collectively, these observations demonstrate that APG-115-mediated p53 activation in macrophages suppresses M2 macrophage polarization and increases M1 macrophage polarization, resulting in a shift from M2 to M1 macrophages.

### APG-115 has co-stimulatory activity in effector T cells

**Fig. 2:**
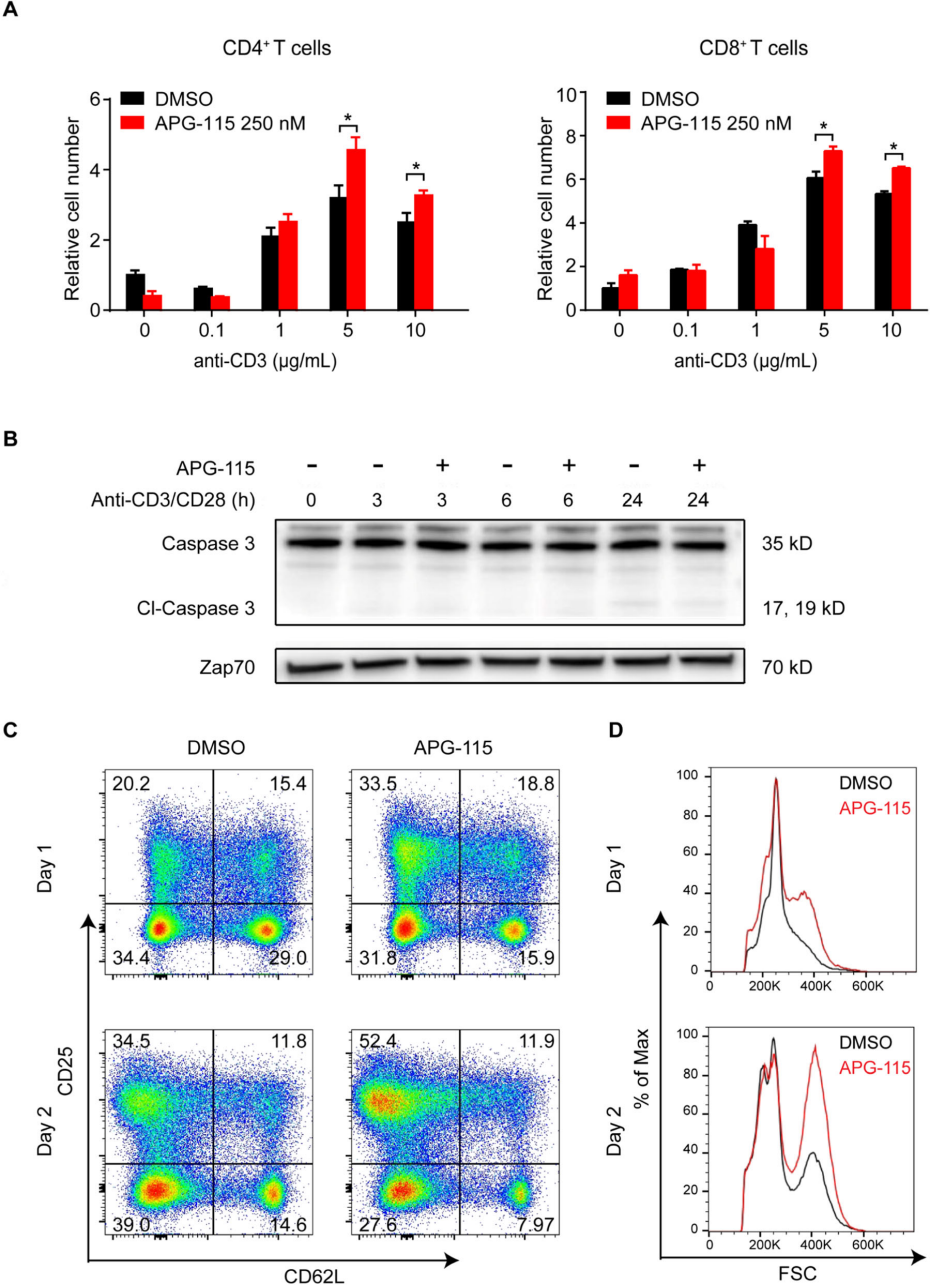
APG-115 increase mouse T cell proliferation and enhances mouse CD4^+^ T cell activation. **a** CD4^+^ T and CD8^+^ T cells were positively selected from mouse spleens using magnetic beads and then stimulated with indicated concentrations of plate-bound anti-CD3 and 2 μg/mL anti-CD28 in the presence of 250 nM APG-115 or DMSO. After 72 h, relative cell numbers were determined using CellTiter-Glo luminescent cell viability assay (Promega) and normalized to unstimulated cultures treated with DMSO control. * *P* < 0.05. **b** immunoblots for the expression of caspase 3, cleaved caspase 3, and Zap-70 (loading control) in total cell lysates of anti-CD3/CD28-stimulated CD4^+^ T cells exposed to APG-115 or solvent control DMSO for 3, 6, or 24 h (h). **c** CD4^+^ T cells were positively selected from mouse spleens using magnetic beads and then stimulated with 10 μg/mL plate-bound anti-CD3 and 2 μg/mL anti-CD28 in the presence of 250 nM APG-115 or DMSO for the indicated periods of time. T cell activation markers (CD25 and CD62L) were determined by flow cytometry. CD25^high^ CD62L^low^ T cells represented an activated population. **d** an increase in cell size was shown after APG-115 treatment

Effector T cells play a critical role in antitumor immunity. Consequently, the effect of MDM2 inhibitor on T cells could impact antitumor immune responses occurring in the context of MDM2 inhibitor-mediated tumor cell death. To investigate how MDM2 inhibitor influences T cells, we exposed CD4^+^ T cells and CD8^+^ T cells isolated from mouse spleens to APG-115 or DMSO control for 72 h. The results showed that APG-115 had a significant effect on T cells, leading to a substantial increase in T cell numbers after 72 h (*P* < 0.05 for 5 and 10 μg/mL, Fig. 2a). This effect was dependent on sufficient stimulation and was not observed under unstimulated or weakly stimulated conditions.

We then investigated whether APG-115 influenced T cell viability and activation. After CD4^+^ T cells isolated from mouse spleens were exposed to 250 nM APG-115 for 3, 6, 24 h, respectively, cleaved caspase 3 was not detected (Fig. 2b). The results indicate that APG-115 at the given concentration does not induce apoptosis of T cells. Interestingly, treatment with 250 nM APG-115 led to a rapid increase in CD25^high^CD62L^low^ cell populations from 20.2 to 33.5% on day 1 and from 34.5 to 52.4% on day 2 (Fig. 2c), as well as an increase in cell size of stimulated CD4^+^ T cells. The results suggest that APG-115 treatment leads to CD4^+^ T cell activation (Fig. 2d).

To exclude the possibility that increased numbers of CD4^+^CD25^+^ cells potentially represented Treg cells, we treated stimulated CD4^+^ T cells with APG-115 for 1 or 2 days and analyzed the potential change in Treg cells (i.e., CD25^+^ and Foxp3^+^). In DMSO-treated cells, an increased percentage of Treg cells was observed after 2 days culture. However, the number of Treg cells remained essentially unchanged in the presence of APG-115, demonstrating that APG-115 dose not selectively expand this population (Additional file 1: Figure S1). The results confirmed CD4^+^ T cell activation under APG-115 treatment. In addition, killing activity of cytotoxic CD8^+^ T cells was not affected by APG-115 (Additional file 2: Figure S2).

### APG-115 upregulates PD-L1 expression on tumor cells

**Fig. 3:**
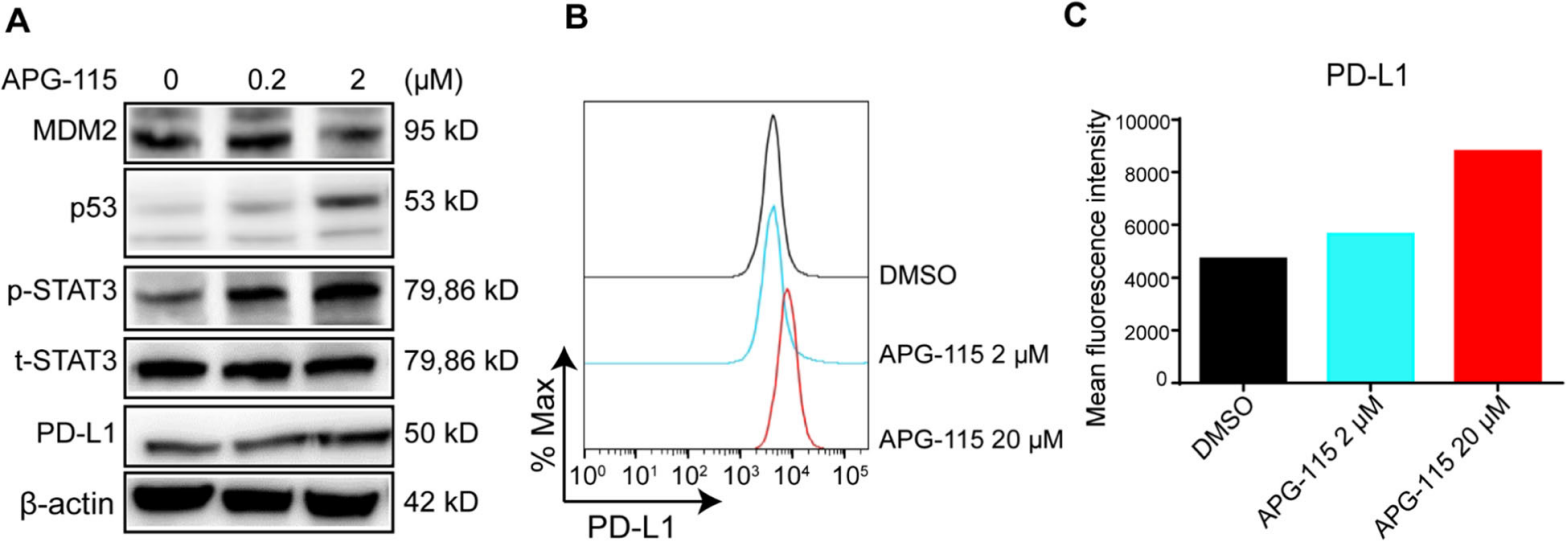
APG-115 upregulates PD-L1 expression on MH-22A tumor cells. MH-22A mouse tumor cells were treated with indicated concentrations of APG-115 for 72 h in vitro. **a** expression levels of MDM2, p53, total STAT3 (t-STAT3), phosphorylated STAT3 (p-STAT3), PD-L1, and β-actin (loading control) were determined by Western blotting. **b** PD-L1 expression levels which were reflected by fluorescence intensity were determined by flow cytometry and the same results were shown as a bar chart (**c**)

Earlier studies suggest that p53 is also involved in the regulation of PD-L1 expression [37]. We then evaluated if APG-115 influenced PD-L1 expression on tumor cells besides its effects on immune cells. After in vitro treatment of MH-22A cells with APG-115, expression of p53 and p-STAT3 proteins were upregulated in a dose-dependent manner, indicating activation of p53 and STAT3 signaling pathway in these tumor cells (Fig. 3a). As a downstream component of STAT3 pathway, PD-L1 levels were elevated accordingly. Flow cytometry analysis further revealed that APG-115 treatment resulted in a dose-dependent increase in the surface expression of PD-L1 on tumor cells (Fig. 3b and c). The data suggests that induction of PD-L1 expression on tumor cells by APG-115 may sensitize these cells to anti-PD-1 therapy.

### APG-115 enhances anti-PD-1-mediated antitumor effect in *Trp53*^*wt*^, *Trp53*^*mut*^, and *Trp53*^*−/−*^ syngeneic mouse tumor models

The above data suggest that p53 activation by APG-115 regulates immune responses, potentially including both adaptive and innate immunity. We then asked if the combined therapy of APG-115 and PD-1 blockade synergistically enhances antitumor immunity in vivo. Presumably, APG-115 activates immune response through the immune cells in the TME and, most likely, its immunological effect is independent of *Trp53* status of tumors. Therefore, syngeneic tumor models with various *Trp53* status, including *Trp53*^*wt*^ MH-22A, *Trp53*^*mut*^ MC38, and *Trp53*^*−/−*^ MH-22A models, were used to test the hypothesis.

**Fig. 4:**
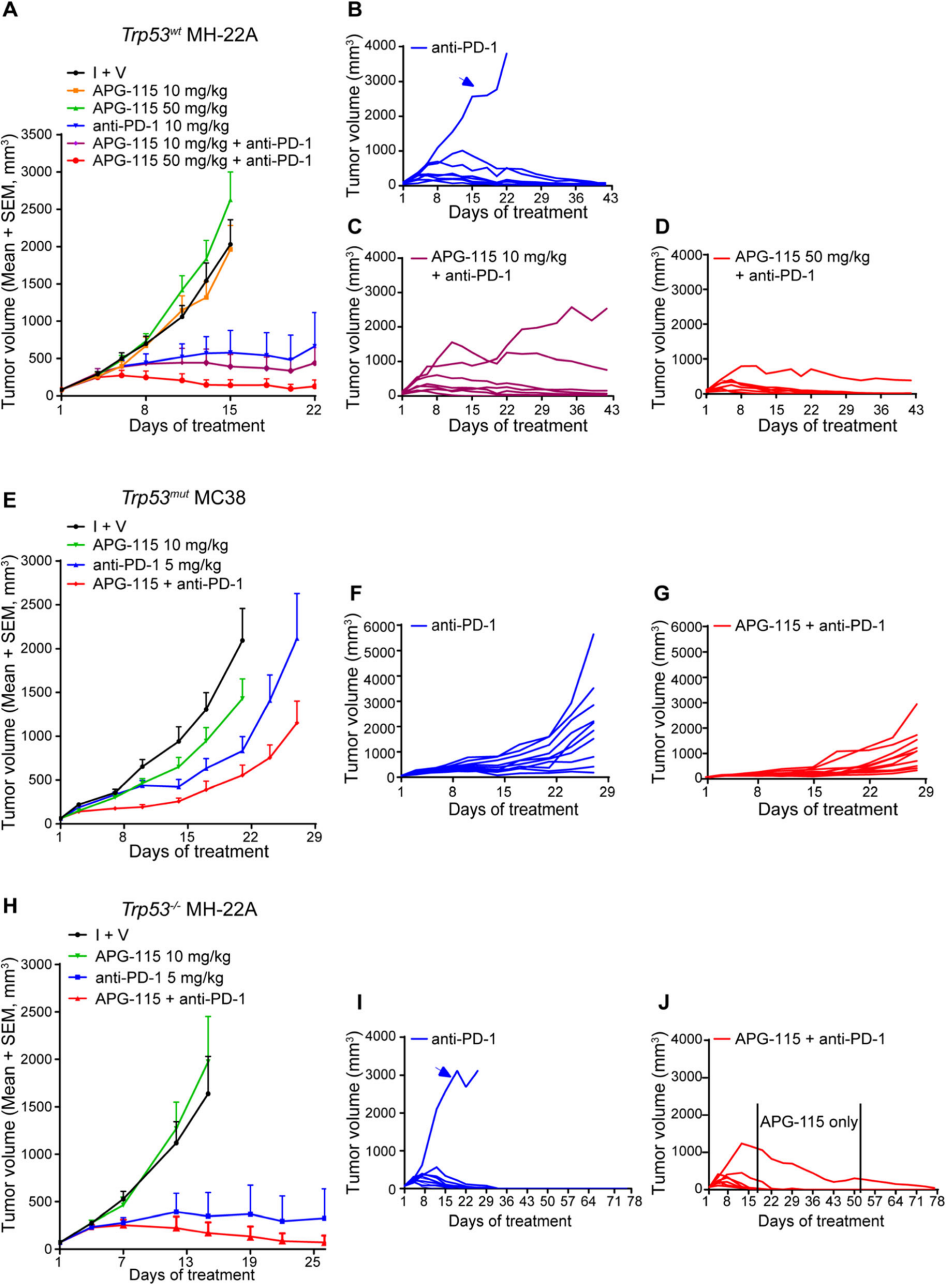
APG-115 enhances anti-PD-1 antibody mediated tumor suppression in *Trp53*^*wt*^, *Trp53*^*mut*^ and *Trp53*^*−/−*^ syngeneic mouse tumor models. APG-115 was tested alone and in combination with anti-PD-1 antibody in mice subcutaneously implanted with ***Trp53***^***wt***^ MH-22A (**a-d;** *n* = 8), ***Trp53***^***mut***^ MC38 (**e-g;** *n* = 10), or ***Trp53***^***−/−***^ MH-22A (**h-j;** n = 10) tumor cells. APG-115 was orally administered every day in *Trp53*^*wt*^ MH-22A models or every other day in both *Trp53*^*mut*^ MC38 and *Trp53*^*−/−*^ MH-22A models. Anti-PD-1 antibody was administered intraperitoneally BIW. Treatments were conducted for 3 weeks in ***Trp53***^***wt***^ MH-22A and ***Trp53***^***mut***^ MC38 models, and for 12 days in ***Trp53***^***−/−***^ MH-22A model. Data representing at least two independent experiments were presented as the mean of tumor volumes of mice in each group (A, E, H) or tumor volumes for individual mice (B, C, D, F, G, I and J). The control groups were treated with APG-115 vehicle (A) or isotype antibody plus vehicle (I + V; E and H)

In *Trp53*^*wt*^ MH-22A hepatoma syngeneic model, APG-115 single agent showed no antitumor activity, whereas the anti-PD-1 antibody effectively reduced tumor volume by exhibiting a T/C (%) value of 22% on d15 (Fig. 4a). Addition of 10 mg/kg or 50 mg/kg APG-115 to PD-1 blockade enhanced antitumor activity by showing T/C (%) values of 17 and 6%, respectively. Because the tumors had reached the maximum allowable size, the animals in vehicle and two APG-115-treated groups were sacrificed on d15 while the remaining three groups continued on treatment. At the end of treatment (d22), one out of eight animals treated with the anti-PD-1 antibody showed SD (i.e., 12.5% response rate). In the combination groups, one SD and one CR appeared under 10 mg/kg APG-115 (i.e., 25% response rate) and one SD and two CR occurred under 50 mg/kg APG-115 treatment (i.e., 37.5% response rate). The tumor growth curves were continually monitored for additional 21 days after drug withdrawal. On d42, the response rates for anti-PD-1, APG-115 (10 mg/kg) plus anti-PD-1, and APG-115 (50 mg/kg) plus anti-PD-1 were 12.5% (1 PR), 25% (2 CR), and 62.5% (2 SD, 1 PR, 2 CR), respectively.

Notably, under treatment with PD-1 single agent, one mouse exhibited progressive disease without showing tumor shrinkage as illustrated in Fig. 4b (arrow). Conversely, the combined therapy was able to delay tumor growth under treatment with 10 mg/kg APG-115 (Fig. 4c) or even convert the resistant tumor into the one responding to the treatment with 50 mg/kg APG-115 (Fig. 4d). These results indicate that the combined therapy enhances antitumor immunity of anti-PD-1 antibody.

Interestingly, in *Trp53*^*mut*^ MC38 murine colon adenocarcinoma model, enhanced antitumor effect was also observed (Fig. 4e). At the end of treatment (d21), T/C (%) values of anti-PD-1 single arm and combination group were 39 and 26%, respectively. The tumor growth rates were substantially delayed in the combination group (Fig. 4f & g).

To confirm the effect of the combined therapy in *Trp53*-deficient tumors, we performed *Trp53* knockout in *Trp53*^*wt*^ MH-22A tumor cells. In comparison with the parental cells, *Trp53* gene was deleted in *Trp53*^*−/−*^ MH-22A cells and, consequently, these cells failed to respond to APG-115 treatment in vitro (Additional file 3: Figure S3). In syngeneic tumor models derived from *Trp53*^*−/−*^ MH-22A cells, the enhanced antitumor effect of the combined therapy was also achieved (Fig. 4h). Specifically, after treatment for 12 days, T/C (%) values in anti-PD-1 single agent and the combination groups were 20.7% (1 SD, 10% response rate) and 10.3% (3 SD, 30% response rate), respectively, on d15. Furthermore, similar to *Trp53*^*wt*^ MH-22A model, one out of 10 animals treated with anti-PD-1 antibody alone exhibited a progressive disease, reaching the maximum allowable tumor volume within 3 weeks (Fig. 4i, arrow). However, in the combined therapy group, tumor growth in all animals was under control, including the animal continually carrying a relatively large tumor (Fig. 4j). Continual monitoring revealed that, the response rates for both anti-PD-1 alone and APG-115 (10 mg/kg) plus anti-PD-1 treatment groups achieved 90% on d78. In fact, there were one SD, one PR and seven CR in the combined therapy group, compared with three SD, one PR and five CR in the anti-PD-1 alone group. The results demonstrated that more CR was achieved by the combined therapy, indicating its stronger antitumor activity, in comparison with anti-PD-1 single agent.

In the animal continually carrying a tumor in the combination group, APG-115 maintenance treatment sustained antitumor effect during d13-d49 (Fig. 4j). On d50, upon regrowth of the tumor, anti-PD-1 therapy was resumed, which led to CR on d78. Together with the data from *Trp53*^*wt*^ MH-22A model (Fig. 4c and d), the results indicate that APG-115 may synergize with anti-PD-1 primarily via a non-tumor cell dependent mechanism.

**Fig. 5:**
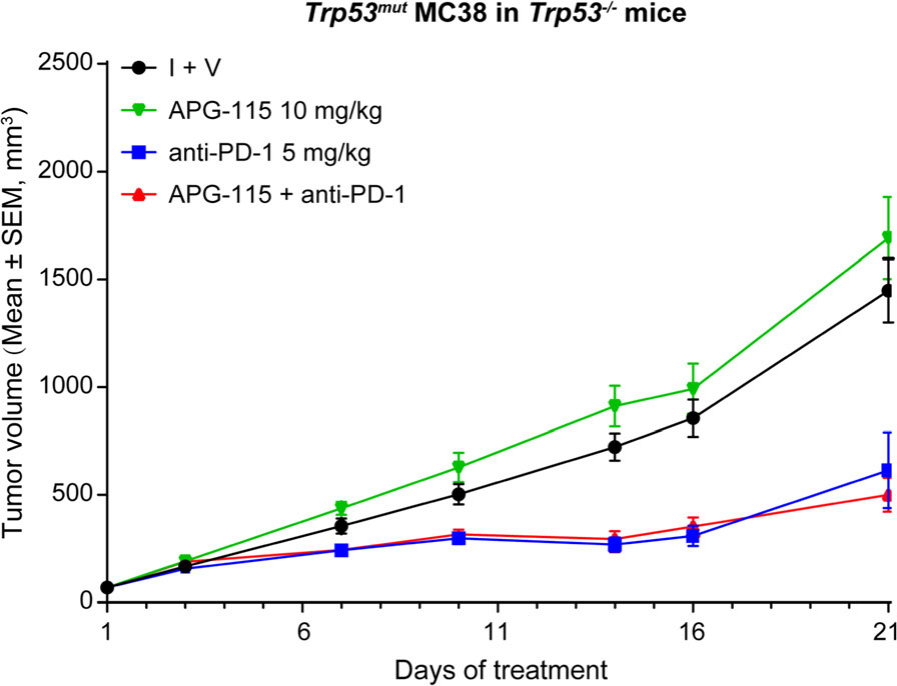
APG-115-enhanced antitumor activity in combination with anti-PD-1 blockade is abolished in *Trp53* knockout mice implanted with *Trp53*^*mut*^ MC38 tumor cells. The effect of APG-115 was evaluated in combination with anti-PD-1 antibody in a subcutaneous MC38 model established in *Trp53* knockout C57BL/6 J mice (*n* = 12/group). APG-115 was orally administered every other day and anti-PD-1 antibody was administered intraperitoneally BIW

To further elucidate the role of the TME in facilitating antitumor activity of the combination therapy, we then asked if the effect persisted in the *Trp53*-knockout mice where host *Trp53* gene was totally deleted. Interestingly, while anti-PD-1 consistently exhibited efficacy, the synergistic effect of the combination therapy was abolished in *Trp53*-knockout mice bearing *Trp53*^*mut*^ MC38 tumor (Fig. 5). The results suggest that an intact p53 in the immune cells of the TME is indispensable for APG-115-mediated efficacious effect in combination with anti-PD-1 therapy.

Notably, the treatments were well tolerated in animals (Additional file 4: Figure S4). Moreover, APG-115 concentrations were examined in the plasma and tumor samples collected from mice bearing *Trp53*^*wt*^ MH-22A tumors (Additional file 5: Figure S5). In the collectable samples, APG-115 concentrations increased dose-proportionally in both plasma and tumor tissues, verifying the correct dosing procedure, as well as appropriate systemic exposure and tissue distribution of APG-115. Furthermore, tumor-free mice after the combination therapy in the *Trp53*^*wt*^ MH-22A study rejected a subsequent injection of MH-22A tumor cells 3 weeks after dosing suspension, suggesting that the animals had successfully developed antitumor immune memory (Additional file 6: Figure S6).

Overall, in the above syngeneic models varying with *Trp53* status of tumors, APG-115 synergizes with PD-1 blockade and the combined therapy demonstrates more profound antitumor activity. Importantly, the effect of APG-115 appears to be independent of *Trp53* status of tumors per se but, instead, requires for wild-type *Trp53* TME.

### APG-115 in combination with PD-1 blockade enhances antitumor immunity in the TME

**Fig. 6:**
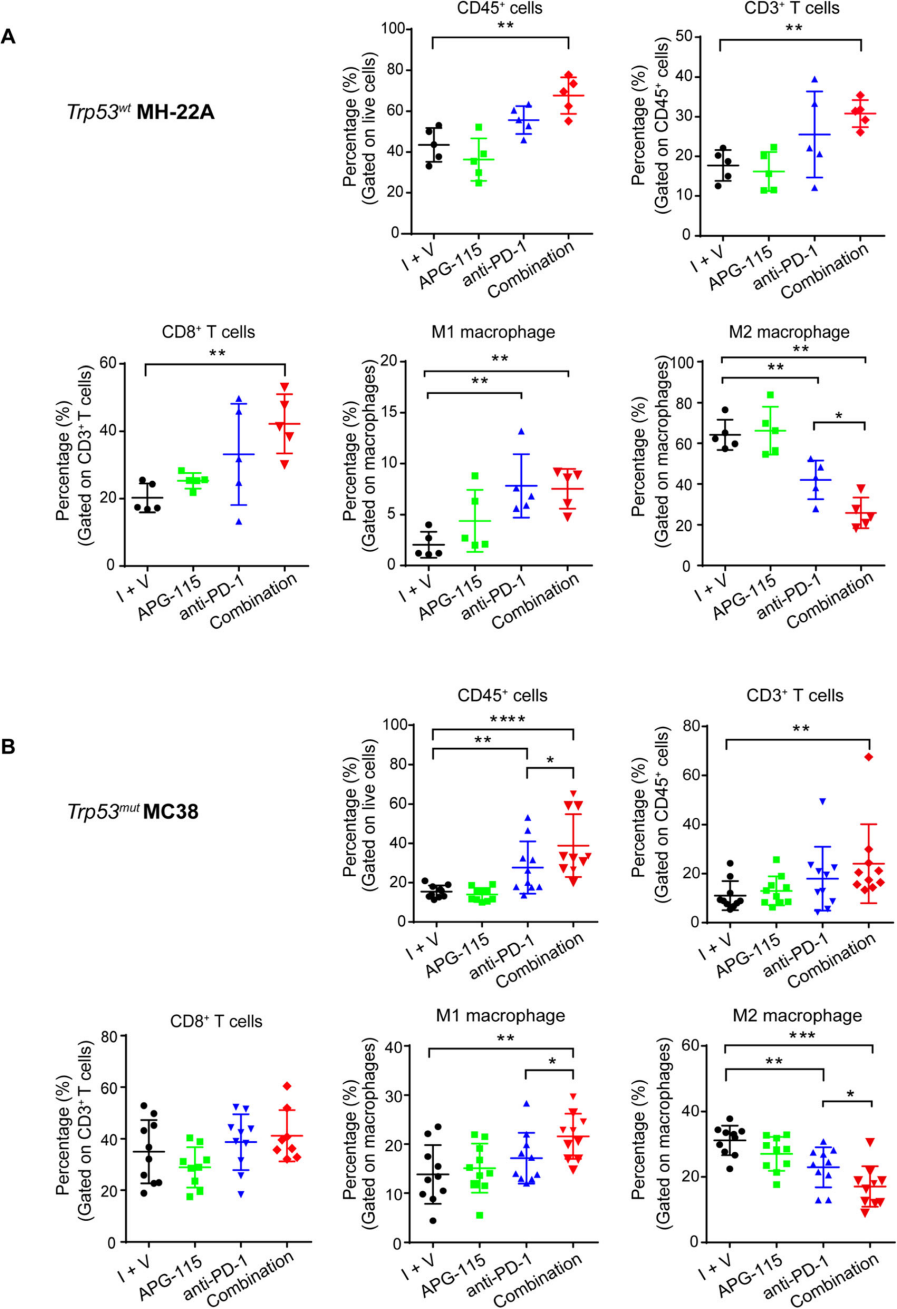
Flow cytometry analysis of TILs in the TME of syngeneic tumors with wild-type (**a**) or mutant (**b**) ***Trp53*.** Mice with established MH-22A or MC38 tumors were treated with 10 mg/kg APG-115 (**a** and **b**), 10 mg/kg (**a**) or 5 mg/kg (**b**) anti-PD-1 antibody, or the combination as described in the legend of Fig. 4. The control group was treated with isotype control antibody and APG-115 vehicle (I + V). On day 14, syngeneic tumors were harvested, dissociated into single-cell suspensions, and stained for flow cytometry analysis. Percentages of CD45^+^, CD3^+^ T cells, CD8^+^ T cells, M1 and M2 macrophages in the tumors under the different treatments were assessed. Data were representative of two (**a**) or three (**b**) independent experiments and shown as dot plots (*n* = 5 or 10). ****P < 0.0001, ***P < 0.001, **P < 0.01, and *P < 0.05, by one-way ANOVA with Bonferroni post-test. I + V indicates isotype control and vehicle of APG-115

To investigate the mechanism underlying enhanced antitumor activity of the combined therapy, we next assessed TILs in the TME by flow cytometry. In *Trp53*^*wt*^ MH-22A syngeneic tumors, in comparison with the control, treatment with anti-PD-1 alone only slightly increased the proportions of CD45^+^ cells, CD3^+^ T cells, and cytotoxic CD8^+^ T cells without reaching statistical significance (*P* > 0.05, Fig. 6a), whereas the combined therapy exerted a more significant effect of increasing infiltration of these cells (*P* < 0.01). There were approximately 1.5 to 2-fold increases relative to the control. Additionally, M1 macrophages was significantly increased by either anti-PD-1 antibody or combined therapy in comparison with the control (*P* < 0.01); however, there was no significant difference between these two treatments (*P* > 0.05). Most strikingly, M2 macrophages was significantly decreased by the combined therapy in comparison with both control (*P* < 0.01) and anti-PD-1 monotherapy (*P* < 0.05).

In *Trp53*^*mut*^ MC38 tumors, in comparison with the control, treatment with anti-PD-1 alone slightly increased the proportions of CD3^+^ T cells, cytotoxic CD8^+^ T cells, and M1 macrophages compared with the control (*P* > 0.05), whereas the frequency of CD45^+^ cells (*P* < 0.001), CD3^+^ T cells (*P* < 0.01), and M1 macrophages (*P* < 0.01), but not CD8^+^ T cells (*P* > 0.05), was significantly increased by the combined therapy (Fig. 6b). Importantly, the proportions of CD45^+^ cells and M1 macrophages were substantially increased by the combined therapy compared with anti-PD-1 monotherapy (*P* < 0.05). In contrast, the frequency of M2 macrophages was remarkably reduced by the combined therapy compared with both control (*P* < 0.001) and anti-PD-1 single agent groups (*P* < 0.05).

In both *Trp53*^*wt*^ MH-22A and *Trp53*^*mut*^ MC38 syngeneic tumors, phenotype analysis of CD4^+^ T cells, NK cells, MDSCs, and regulatory T (Treg) cells showed no significant changes after treatment with APG-115, anti-PD-1 antibody, or the combination (Additional file 7: Figure S7). In addition to assessing the proportions of CD4^+^ and CD8^+^ T cells between different treatment groups, we analyzed the levels of IFN-γ and TNF-α in T cells in MH-22A model. A significant increase in the proportion of CD4^+^ T cells expressing IFN-γ under combination treatment with APG-115 and anti-PD-1 was observed in comparison with the vehicle control (*P* < 0.0001) and anti-PD-1 monotherapy (*P* < 0.0001) (Additional file 8: Figure S8). No changes were observed in the fraction of CD4^+^ T expressing TNF-α or CD8^+^ T cells expressing IFN-γ and TNF-α. In consistent with our in vitro findings, these results demonstrate that APG-115 enhance the activation of CD4^+^ T cells while has no effects on cytotoxic activity of CD8^+^ T cells.

Taken together, the combination treatment significantly improves cytotoxic CD8^+^ T cell infiltration in the TME of *Trp53*^*wt*^ tumors, as well as M1 macrophage infiltration in the TME of *Trp53*^*mut*^ tumors. Most importantly, the combined therapy consistently reduces immunosuppressive M2 macrophages in both *Trp53*^*wt*^ and *Trp53*^*mut*^ tumors. These results demonstrate that the combination treatment reverses the immunosuppressive TME into antitumor immunity, leading to enhanced therapeutic benefit in mice.

## Discussion

The TME of human tumors is composed of blood vessels, fibroblasts, immune cells, signaling molecules and the extracellular matrix. Successful development of tumors and subsequent metastasis is driven by not only genetic or epigenetic alterations in tumor cells, but also protumoral TME. Macrophages present abundantly in the TME of most tumor types and high infiltration of TAMs is associated with poor prognosis and contributes to chemotherapy resistance [19] .

PD-(L)1 blockade therapy potentiates the activity of cytotoxic CD8^+^ T cells and has demonstrated clinical benefits in multiple cancer types. However, only a small subpopulation of patients responds to immunotherapy due to various reasons, including the immunosuppressive TME. MDM2 amplification has been suggested as a potential mechanism for hyperprogressive disease developed in some patients after immunotherapy, raising the possibility that a combination strategy with MDM2 inhibitor could limit hyperprogression on immunotherapy [3]. Moreover, tumor suppressor p53 plays a critical role in immune modulation [38]. Particularly, p53 activation in the myeloid lineage influences the innate immune response by reprograming M2 to M1 macrophages to suppress tumorigenesis [30]. Local activation of p53 by a MDM2 inhibitor nutlin-3a in *Trp53*^*wt*^ syngeneic tumors is able to reverse immunosuppressive to immunostimulatory TME and exert antitumor immunity [39]. Overall, reversing the immunosuppressive TME has become one of the promising therapeutic strategies to improve immunotherapy.

In our study, we applied a clinical-stage MDM2 inhibitor APG-115 to targeting MDM2-p53 pathway in order to study the role of p53 activation in immune modulation and search for an enhancer of immunotherapy. Collectively, our results demonstrate that, in *Trp53*^*mut*^ tumors, the combination of APG-115 and PD-1 blockade promotes an antitumor immunity through downregulation of immunosuppressive M2 macrophages. In *Trp53*^*wt*^ tumors, the combined therapy not only reduces the fraction of M2 macrophages, but also synergistically induces more significant infiltration of cytotoxic CD8^+^ T cells in the TME. Consistently, in vitro, APG-115 single agent suppresses alternative (M2) macrophages polarization and increases M1 macrophages, which is mediated by downregulation of c-Myc and c-Maf through p53 activation in these immune cells. Collectively, both adaptive and innate antitumor responses are activated by APG-115 in *Trp53*^*wt*^ tumors; however, innate antitumor immunity seems to play a primary role in *Trp53*^*mut*^ tumors treated with APG-115. Considering that tissue resident macrophages suppress CD4^+^ and CD8^+^ T cell proliferation and cytokine production [40], the switch from M2 to M1 macrophages may also indirectly promote adaptive antitumor immunity in *Trp53*^*mut*^ tumors. Most importantly, our results demonstrate that APG-115-stimulated immunity is able to sensitize resistant tumors to PD-1 blockade into sensitive tumors and such a therapy approach may apply to both *Trp53*^*wt*^ and *Trp53*^*mut*^ tumors, creating a significant impact because approximately 50% of human cancers are p53 dysfunctional or mutant [41].

Additionally, APG-115 single agent increases T cell numbers in the presence of anti-CD3/CD28 antibodies, and enhances mouse CD4^+^ T cell activation. APG-115 appears to regulate immune cells via modulation of p53 activation as well as affect tumor cells, because APG-115 treatment led to increased PD-L1 expression in tumor cells. Further studies are warranted to more closely examine the immunological regulation by APG-115 on both tumor and infiltrating immune cells.

Enhanced antitumor effect of MDM2 inhibitors in combination with immunotherapy has been recently reported [42, 43]. Increased numbers of CD103^+^ DC cells, Tbet^+^ EOMES^−^ T cells, and ratios of CD8^+^ T cells/Treg were observed with a MDM2 inhibitor NVP-HDM201 treatment in murine tumors as well as tumor draining lymph nodes, leading to synergistic effect of NVP-HDM201 in combination with anti-PD-1 or PD-L1 antibody in syngeneic tumor models [42]. Similarly, synergistic efficacy was observed in combination treatment with another MDM2 inhibitor BI907828 and anti-PD-1 antibody in a syngeneic tumor model. Mechanistically, CD8^+^ T cells, but not CD4^+^ T cells, were required to achieve tumor regression [43]. Both reports stated that the synergistic effect was observed only in *Trp53*^*wt*^ tumors. Consistent with these reports, in *Trp53*^*wt*^ tumors, we also demonstrate substantial antitumor effect of the combination of APG-115 and anti-PD-1 antibody, together with a significant increase in infiltrated cytotoxic CD8^+^ T cells, implicating for the importance of CD8^+^ T cell-mediated antitumor immunity in *Trp53*^*wt*^ tumors. Furthermore, for the first time, our studies revealed that APG-115 enables reprogramming of M2 macrophages and promotes antitumor immunity in not only *Trp53*^*wt*^, but also *Trp53*^*mut*^ tumors. Presumably, the effect of APG-115 is facilitated by wild-type immune cells in the TME through p53 activation. Further investigation is required to fully understand the mechanism underlying the differential immune responses elicited by APG-115-mediated p53 activation in the TME between *Trp53*^*wt*^ and *Trp53*^*mut*^ tumors.

Although the above two presentations concluded that immune checkpoint inhibitor in combination with MDM2 inhibitor only worked synergistically in *Trp53*^*wt*^ tumors, no comprehensive studies of the combinatorial effect were described in *Trp53*^*mut*^ tumors [42, 43]. Therefore, it is difficult for us to interpret their results without evaluating the results of these two MDM2 inhibitors in *Trp53*^*mut*^ tumor models. Considering the antitumor activity of the combination in *Trp53*^*mut*^ tumors in comparison with *Trp53*^*wt*^ tumors, most likely due to lack of the increase in infiltrated cytotoxic CD8^+^ T cells in the TME, the effect of the combination treatment might be neglected. In fact, in the poster presentation of BI907828 [42, 43], the antitumor activity of the MDM2 inhibitor had been seen to a certain degree in *Trp53*^*mut*^ MC38 syngeneic tumors in C57BL/6 mice. But the authors then turned to *Trp53*^*wt*^ Colon-26 syngeneic tumor models to demonstrate the synergy with an immune checkpoint inhibitor. Therefore, it will be more critical to simultaneously evaluate the effect of MDM2 inhibitors on the TILs in both *Trp53*^*mut*^ and *Trp53*^*wt*^ syngeneic tumor models before a conclusion is drawn.

In genetically engineered mouse models, it has been demonstrated that p53 activation within the myeloid lineage of the TME is capable of suppressing M2 macrophage polarization and inhibiting tumor growth and progression [30]. Combining macrophage-modulating agents and immune checkpoint blockade makes sense and has emerged as attractive therapeutic goals in the treatment of cancer, especially in the context of tumors that are enriched with immunosuppressive macrophages. For example, it was recently demonstrated that antibody targeting of MARCO-expressing TAMs blocked tumor growth and metastasis, and also enhanced the effects of immune checkpoint therapy in melanoma and colon carcinoma models [44]. Targeting legumain, a highly overexpressed target molecule on M2 macrophage effectively decreased the release of protumoral growth and angiogenic factors, which in turn, led to suppression of both tumor angiogenesis, tumor growth and metastasis [45]. Similarly, the supramolecule blocking the CD47-SIRPα signaling axis while sustainably inhibiting CSF-1R enhanced M2 to M1 repolarization within the TME and significantly improved antitumor and antimetastatic efficacies in animal models of melanoma and breast cancer [46].

It is worth noting that, the dosing levels of a MDM2 inhibitor required for p53 activation in the cellular compartment of the TME and reversal of immunosuppression are well below the dose levels for exerting direct tumoricidal activity against tumor cells [39]. In our study, we found that 10 mg/kg APG-115 in mice was sufficient to trigger antitumor immunity in the TME through p53 activation. The clinically relevant dose of 10 mg/kg in mice was approximately 50 mg daily in humans, which was well tolerated in our clinical trials (data not shown). Furthermore, based on the systemic exposure data of APG-115, the corresponding dose level of 10 mg/kg in vivo is approximately 250 nM in vitro and no induction of apoptosis was observed in immune cells at such a concentration. Therefore, we anticipate that the combined therapy will be safe for patients.

## Conclusion

Collectively, our results suggest that APG-115 enhances antitumor immunity in combination with PD-1 blockade through activation of both adaptive and innate immunity in *Trp53*^*wt*^ tumors. In *Trp53*^*mut*^ tumors, the enhanced effect of APG-115 is mainly mediated by innate immunity through the shift of M2 macrophages into M1 macrophages in the TME. In *Trp53*^*wt*^ tumors, in addition to the M2/M1 shift of macrophages, enhanced CD4^+^ T cell function and elevated cytotoxic CD8^+^ T cell infiltration may jointly contribute to the enhanced activity.

APG-115 as a single agent plays multiple roles in modulating immune responses, including increasing T cell proliferation, enhancing CD4^+^ T cell activation, upregulating PD-L1 expression on tumor cells, and increasing M1 macrophages either in vitro or in vivo. These data indicate that MDM2 inhibition acts as an important immune regulator in the tumor microenvironment. Accordingly, in combination treatment, MDM2 inhibitor APG-115 improves the efficacy of anti-PD-1 therapy. Importantly, the synergistic effect of the combined therapy is independent of the *Trp53* status of tumors per se because APG-115 primarily regulates the immune compartments of the TME through p53 activation. Furthermore, the immune compartment activated by APG-115 appears to be complementary to that by anti-PD-(L)1 therapy.

Based on the promising preclinical data, we have initiated a phase 1b clinical trial to evaluate the synergistic effect of APG-115 in combination with pembrolizumab in patients with solid tumors (NCT03611868).

## Supplementary information

**Additional file 1: Figure S1** APG-115 does not selectively expand Treg population. CD4^+^ T cell were positively selected from mouse spleens using magnetic beads and then stimulated with 10 μg/mL plate-bound anti-CD3 and 2 μg/mL anti-CD28 in the presence of 250 nM APG-115 or solve control DMSO for the indicated periods of time. Regulatory T cell (Treg) markers (CD25 and Foxp3) were determined by flow cytometry. CD25^+^Foxp3^+^ T cells represented Treg population.

**Additional file 2: Figure S2** APG-115 does not affect cytotoxic activity of CD8^+^ T cells. The effect of APG-115 on cytotoxic activity of CD8^+^ T cells was assessed as described in detail in the Materials and Methods section. Percentage of target cell lysis were presented.

**Additional file 3: Figure S3** Upon knockout of *Trp53* gene, *Trp53*^*−/−*^ MH-22A tumor cells fail to respond to APG-115 treatment. Both *Trp53*^*wt*^ and *Trp53*^*−/−*^ MH-22A tumor cells were treated with APG-115 (4 μM) for 24 h. The expression levels of total protein p53, p21 and β-actin (loading control) were determined by Western blotting.

**Additional file 4: Figure S4** No significant loss of body weights in mice treated with the combined therapy. Percentage change of the body weight of animals in the experiments of *Trp53*^*wt*^ MH-22A tumor (**A**), *Trp53*^*mut*^ MC38 tumor (**B**) and *Trp53*^*−/−*^ MH-22A tumors (**C**). I + V indicates isotype control and vehicle of APG-115.

**Additional file 5: Figure S5** Mean plasma and tumor concentrations of APG-115 in MH-22A tumor bearing mice after treatment. Mice bearing MH-22A tumor were treated with vehicle, APG-115, anti-PD-1 alone or their combination. Four hours after the drug administration on day eight, the plasma and tumor concentrations of APG-115 were analyzed by quantitative liquid chromatography mass spectrometry (LC/MS/MS). Briefly, quantitative LC/MS/MS analysis was conducted using an Exion HPLC system (AB Sciex) coupled to an API 5500 mass spectrometer (AB Sciex) equipped with an API electrospray ionization source. The Phenomenex Titank phenyl-Hexyl column (50 mm × 2.1 mm, 5 μm particle size) was used to achieved HPLC separation. The injection volume was 2 μL and the flow rate was kept constantly at 0.5 mL/min. Chromatography was performed with mobile phase A, acetonitrile: water: formic (5:95:0.1, in volume) and B, acetonitrile: water: formic (95:5:0.1, in volume). The mass spectrometer was operated at ESI positive ion mode for APG-115. The results were presented as dot plots with each dot representing a sample.

**Additional file 6: Figure S6** CR mice cured by the combined therapy develop immune memory against tumor antigens expressed in the MH-22A tumor. There were totally eight tumor-bearing mice exhibiting CR after the combined therapy with APG-115 plus anti-PD-1 antibody (Fig. 4a). To assess immune memory, these animals were re-challenged by inoculating murine MH-22A liver tumor cells 3 weeks post the last treatment as detailed in the Materials and Methods section. Naïve C3H mice were inoculated with the tumor cells as the control. The tumor growth curves of the pooled (A) and individual mice (B and C) were presented.

**Additional file 7: Figure S7** Flow cytometry analysis of CD4^+^ T cells, NK cells, MDSC and Treg cells in the TME of syngeneic tumors with wild-type (A, MH-22A) or mutant (B, MC38) *Trp53*. Besides the frequency of TILs shown in Fig. 5, additional ones were shown here (*n* = 5 or 10). I + V indicates isotype control and vehicle of APG-115.

**Additional file 8: Figure S8** Combined treatment with APG-115 and anti-PD-1 increases tumor infiltrated CD4^+^ IFN-γ^+^ T cells. Mice with established MH-22A tumors were treated with APG-115 and anti-PD-1 as described in the legend of Fig. 4. Tumors were isolated on day seven after the first treatment and the expression levels of IFN-γ, TNF-α in T cells were analyzed by flow cytometry (*n* = 10/group). Shown are percentages of IFN-γ^+^, TNF-α^+^ within CD4^+^ T cells (A), and within CD8^+^ T cells (B). *P < 0.05 and ****P < 0.0001, by one-way ANOVA followed with Turkey’s multiple comparisons test. I + V indicates isotype control and vehicle of APG-115.

## Data Availability

The datasets used and/or analyzed during the current study are available from the corresponding author on reasonable request.

## Abbreviations

BIW: Twice a week
BMDM: Bone-marrow derived macrophage\
CR: Complete regression
HPLC: High-performance liquid chromatography
HPMC: Hydroxypropyl methylcellulose
IFN-γ: Gamma-interferon
IL: Interleukin
m-CSF: Macrophage colony stimulating factor
MDM2: Mouse double minute 2 homolog
MDSC: Myeloid-derived suppressor cells
Mut: Mutant
PBS: Phosphate buffered saline
PR: Partial tumor regression
RECIST: Response evaluation criteria in solid tumors
RTV: Relative tumor volume
SD: Stable disease
T/C (%): T_RTV_/C_RTV_ × 100%
TAM: Tumor associated macrophage
TIL: Tumor infiltrating lymphocytes
TME: Tumor microenvironment
TNF-α: Tumor necrosis factor α
Treg: Regulatory T cells
WT: Wild-type
