# Supplementary material for: MDM2 inhibitor APG-115 synergizes with PD-1 blockade through enhancing antitumor immunity in the tumor microenvironment
Source: J Immunother Cancer. 2019 Nov 28;7:327. doi: 10.1186/s40425-019-0750-6 (PMC6883539; doi:10.1186/s40425-019-0750-6)
Supplement: Supplementary file 1 — Additional file 1: Figure S1 APG-115 does not selectively expand Treg population. CD4+ T cell were positively selected from mouse spleens using magnetic beads and then stimulated with 10 μg/mL plate-bound anti-CD3 and 2 μg/mL anti-CD28 in the presence of 250 nM APG-115 or solve control DMSO for the indicated periods of time. Regulatory T cell (Treg) markers (CD25 and Foxp3) were determined by flow cytometry. CD25+Foxp3+ T cells represented Treg population. [file 40425_2019_750_MOESM1_ESM.docx]

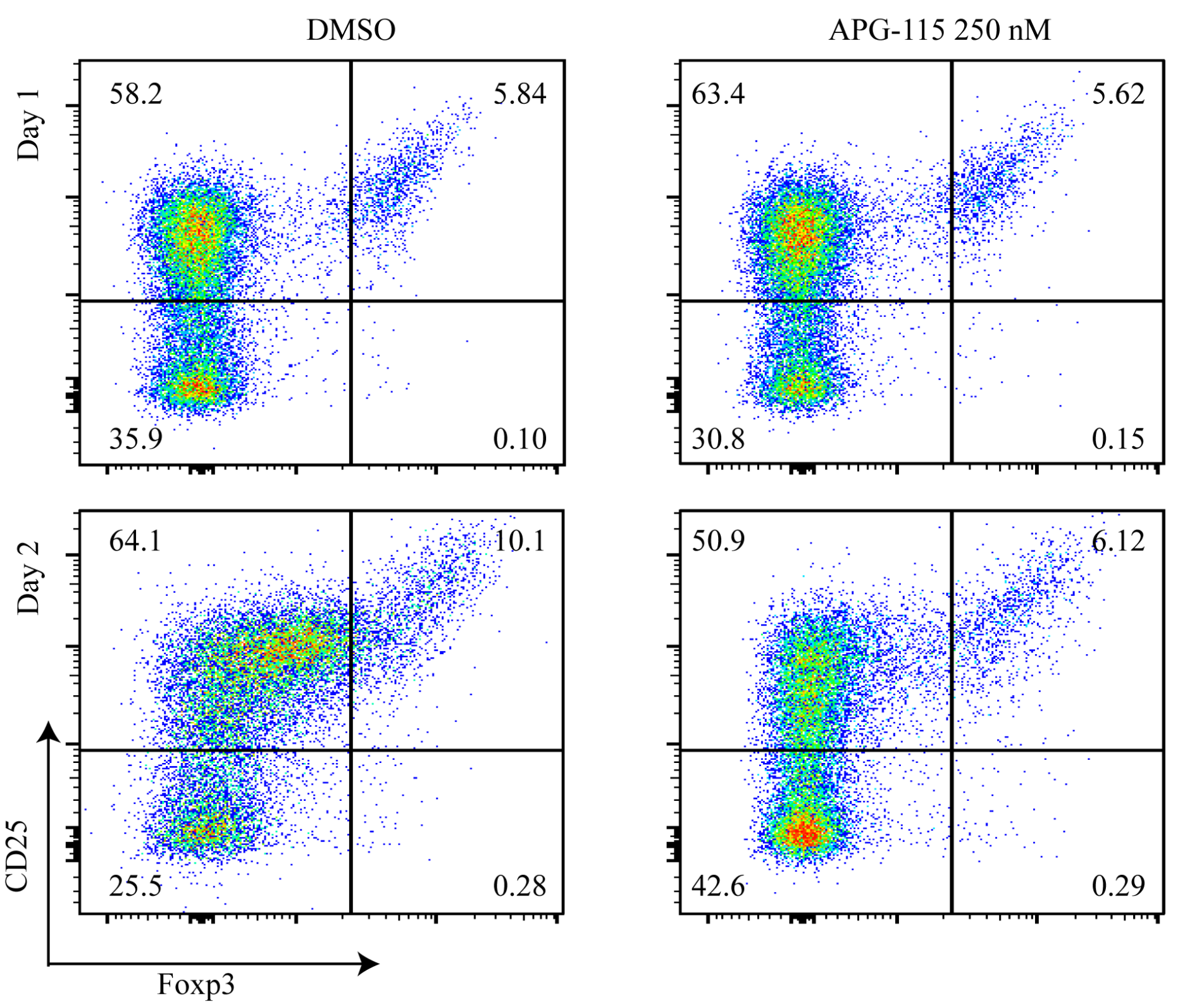


**Figure S1. APG-115 does not selectively expand Treg population.**

CD4^+^ T cell were positively selected from mouse spleens using magnetic beads and then stimulated with 10 μg/mL plate-bound anti-CD3 and 2 μg/mL anti-CD28 in the presence of 250 nM APG-115 or solve control DMSO for the indicated periods of time. Regulatory T cell (Treg) markers (CD25 and Foxp3) were determined by flow cytometry. CD25^+^Foxp3^+^ T cells represented Treg population.
