# Supplementary material for: MDM2 inhibitor APG-115 synergizes with PD-1 blockade through enhancing antitumor immunity in the tumor microenvironment
Source: J Immunother Cancer. 2019 Nov 28;7:327. doi: 10.1186/s40425-019-0750-6 (PMC6883539; doi:10.1186/s40425-019-0750-6)
Supplement: Supplementary file 2 — Additional file 2: Figure S2 APG-115 does not affect cytotoxic activity of CD8+ T cells. The effect of APG-115 on cytotoxic activity of CD8+ T cells was assessed as described in detail in the Materials and Methods section. Percentage of target cell lysis were presented. [file 40425_2019_750_MOESM2_ESM.docx]

**
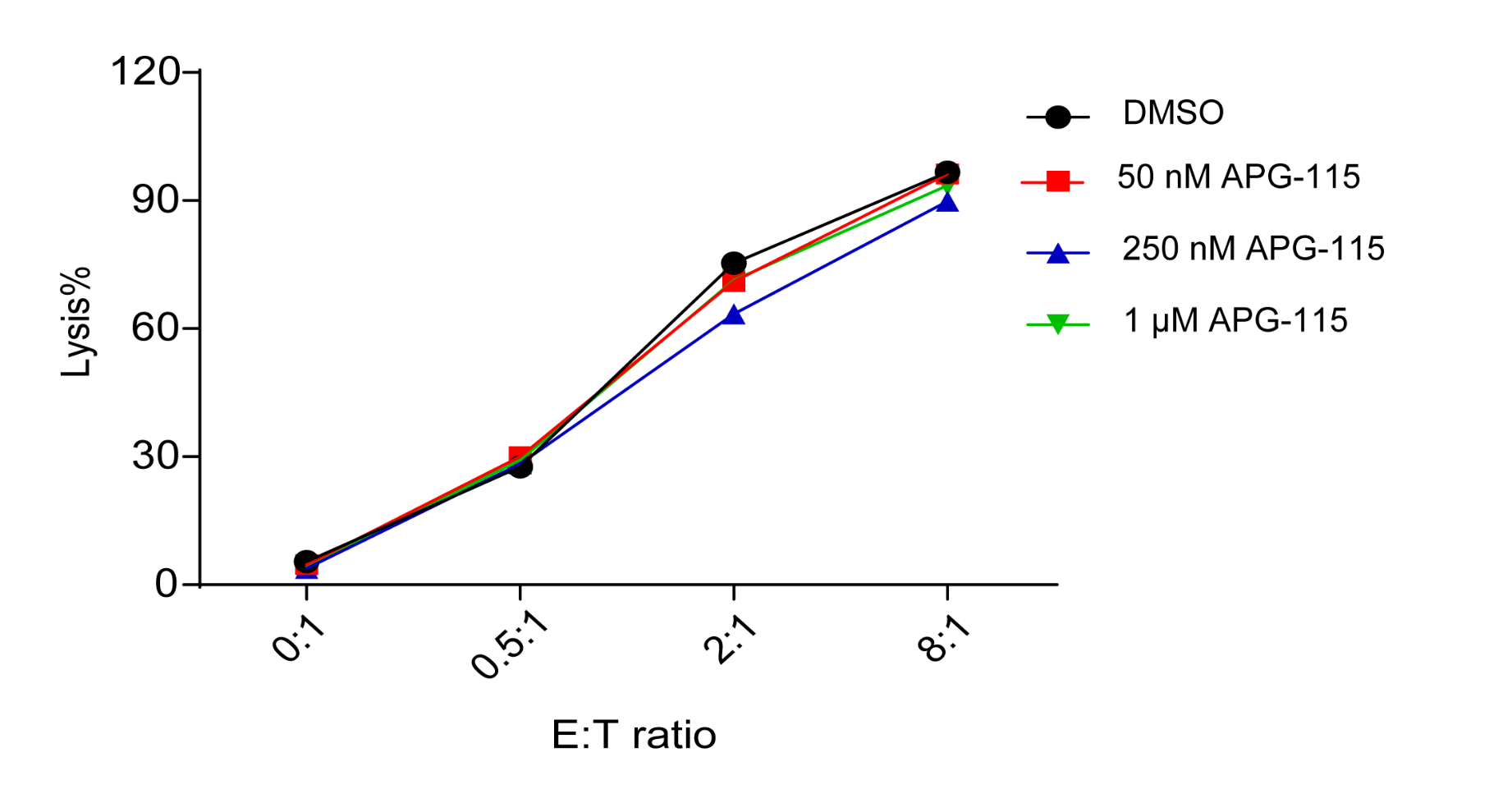
**

**Figure S2.** **APG-115 does not affect cytotoxic activity of CD8^+^ T cells.** The effect of APG-115 on cytotoxic activity of CD8^+^ T cells was assessed as described in detail in the Materials and Methods section. Percentage of target cell lysis were presented.
