# Supplementary material for: MDM2 inhibitor APG-115 synergizes with PD-1 blockade through enhancing antitumor immunity in the tumor microenvironment
Source: J Immunother Cancer. 2019 Nov 28;7:327. doi: 10.1186/s40425-019-0750-6 (PMC6883539; doi:10.1186/s40425-019-0750-6)
Supplement: Supplementary file 3 — Additional file 3: Figure S3 Upon knockout of Trp53 gene, Trp53−/− MH-22A tumor cells fail to respond to APG-115 treatment. Both Trp53wt and Trp53−/− MH-22A tumor cells were treated with APG-115 (4 μM) for 24 h. The expression levels of total protein p53, p21 and β-actin (loading control) were determined by Western blotting. [file 40425_2019_750_MOESM3_ESM.docx]

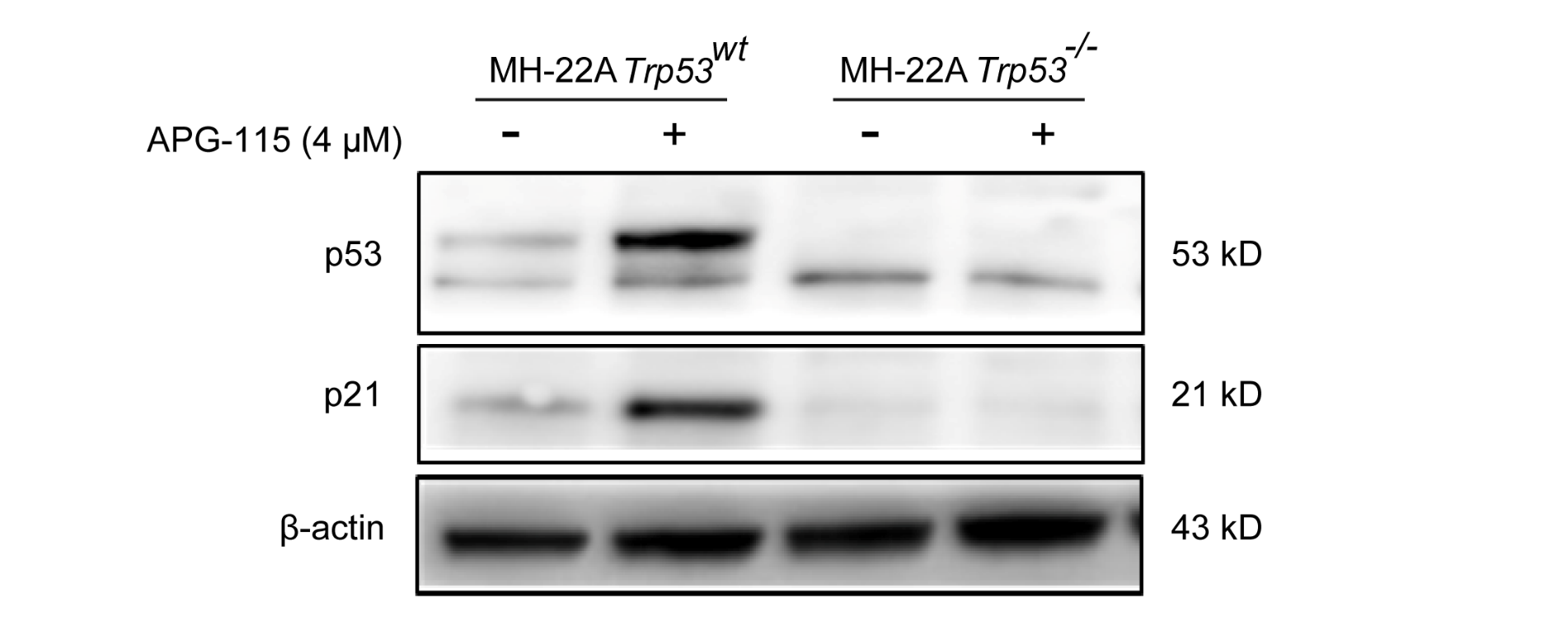


**Figure S3. Upon knockout of *Trp53* gene, *Trp53^-/-^* MH-22A tumor cells fail to respond to APG-115 treatment.** Both *Trp53^wt^* and *Trp53^-/-^* MH-22A tumor cells were treated with APG-115 (4 μM) for 24 hours. The expression levels of total protein p53, p21 and β-actin (loading control) were determined by Western blotting.
