# Supplementary material for: MDM2 inhibitor APG-115 synergizes with PD-1 blockade through enhancing antitumor immunity in the tumor microenvironment
Source: J Immunother Cancer. 2019 Nov 28;7:327. doi: 10.1186/s40425-019-0750-6 (PMC6883539; doi:10.1186/s40425-019-0750-6)
Supplement: Supplementary file 4 — Additional file 4: Figure S4 No significant loss of body weights in mice treated with the combined therapy. Percentage change of the body weight of animals in the experiments of Trp53wt MH-22A tumor (A), Trp53mut MC38 tumor (B) and Trp53−/− MH-22A tumors (C). I + V indicates isotype control and vehicle of APG-115. [file 40425_2019_750_MOESM4_ESM.docx]

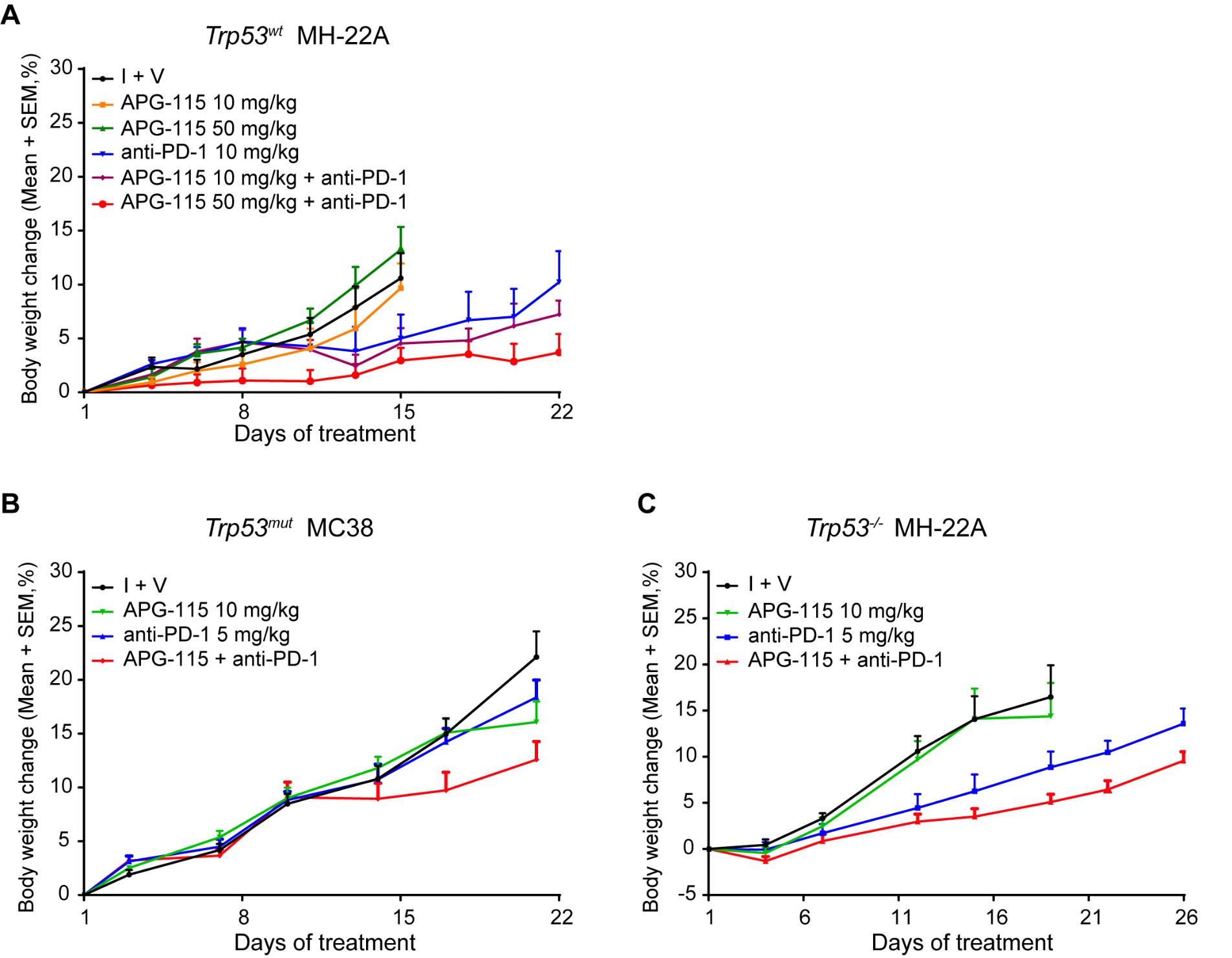


**Figure S4. No significant loss of body weights in mice treated with the combined therapy.** Percentage change of the body weight of animals in the experiments of *Trp53^wt^* MH-22A tumor (**A**), *Trp53^mut^* MC38 tumor (**B**) and *Trp53^-/-^* MH-22A tumors (**C**). I + V indicates isotype control and vehicle of APG-115.
