# Supplementary material for: MDM2 inhibitor APG-115 synergizes with PD-1 blockade through enhancing antitumor immunity in the tumor microenvironment
Source: J Immunother Cancer. 2019 Nov 28;7:327. doi: 10.1186/s40425-019-0750-6 (PMC6883539; doi:10.1186/s40425-019-0750-6)
Supplement: Supplementary file 5 — Additional file 5: Figure S5 Mean plasma and tumor concentrations of APG-115 in MH-22A tumor bearing mice after treatment. Mice bearing MH-22A tumor were treated with vehicle, APG-115, anti-PD-1 alone or their combination. Four hours after the drug administration on day eight, the plasma and tumor concentrations of APG-115 were analyzed by quantitative liquid chromatography mass spectrometry (LC/MS/MS). Briefly, quantitative LC/MS/MS analysis was conducted using an Exion HPLC system (AB Sciex) coupled to an API 5500 mass spectrometer (AB Sciex) equipped with an API electrospray ionization source. The Phenomenex Titank phenyl-Hexyl column (50 mm × 2.1 mm, 5 μm particle size) was used to achieved HPLC separation. The injection volume was 2 μL and the flow rate was kept constantly at 0.5 mL/min. Chromatography was performed with mobile phase A, acetonitrile: water: formic (5:95:0.1, in volume) and B, acetonitrile: water: formic (95:5:0.1, in volume). The mass spectrometer was operated at ESI positive ion mode for APG-115. The results were presented as dot plots with each dot representing a sample. [file 40425_2019_750_MOESM5_ESM.docx]

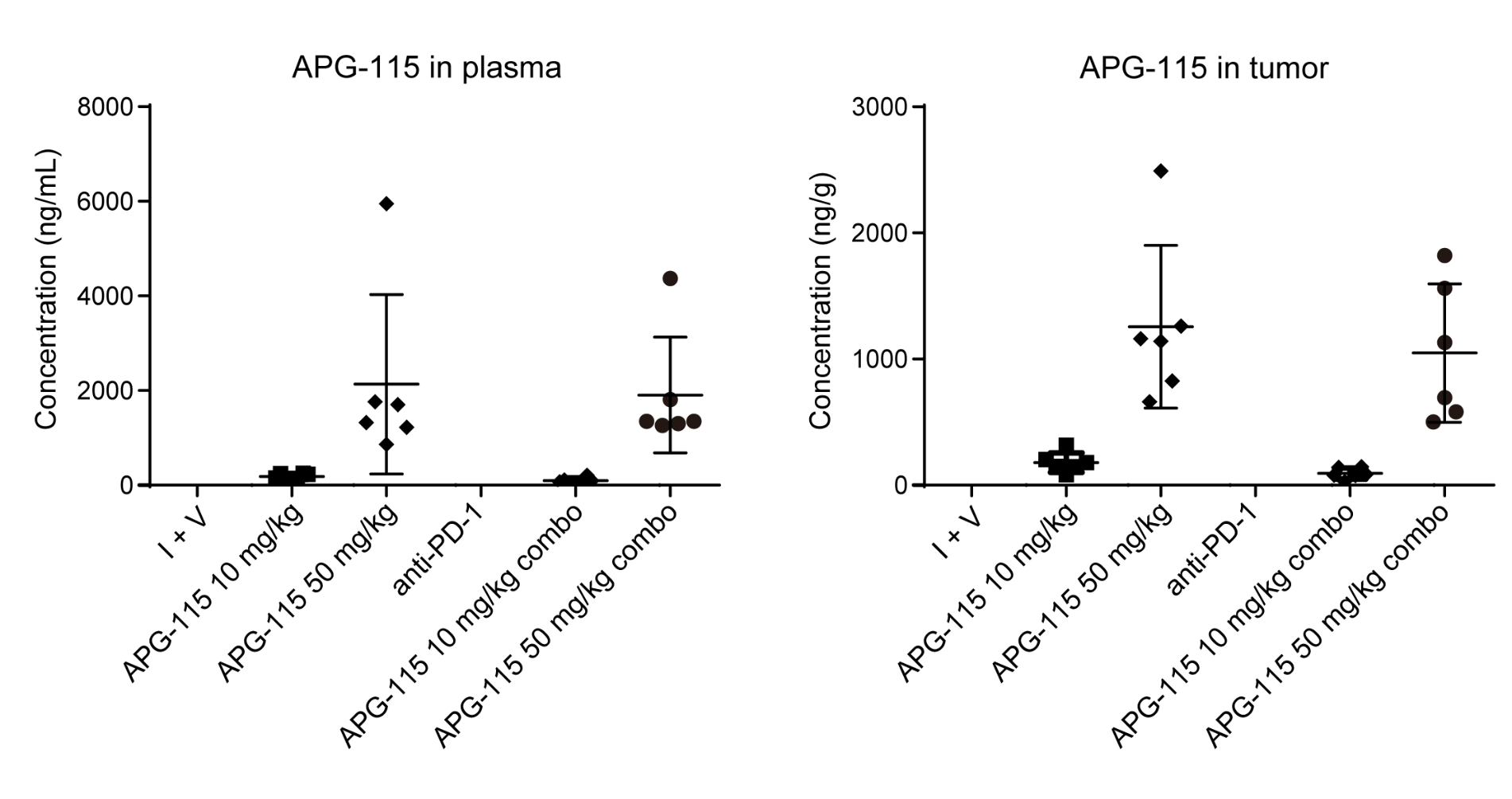


**Figure S5. Mean plasma and tumor concentrations of APG-115 in MH-22A tumor bearing mice after treatment.** Mice bearing MH-22A tumor were treated with vehicle, APG-115, anti-PD-1 alone or their combination. Four hours after the drug administration on day eight, the plasma and tumor concentrations of APG-115 were analyzed by quantitative liquid chromatography mass spectrometry (LC/MS/MS). Briefly, quantitative LC/MS/MS analysis was conducted using an Exion HPLC system (AB Sciex) coupled to an API 5500 mass spectrometer (AB Sciex) equipped with an API electrospray ionization source. The Phenomenex Titank phenyl-Hexyl column (50 mm × 2.1 mm, 5 μm particle size) was used to achieved HPLC separation. The injection volume was 2 µL and the flow rate was kept constantly at 0.5 mL/min. Chromatography was performed with mobile phase A, acetonitrile: water: formic (5:95:0.1, in volume) and B, acetonitrile: water: formic (95:5:0.1, in volume). The mass spectrometer was operated at ESI positive ion mode for APG-115. The results were presented as dot plots with each dot representing a sample.
