# Supplementary material for: MDM2 inhibitor APG-115 synergizes with PD-1 blockade through enhancing antitumor immunity in the tumor microenvironment
Source: J Immunother Cancer. 2019 Nov 28;7:327. doi: 10.1186/s40425-019-0750-6 (PMC6883539; doi:10.1186/s40425-019-0750-6)
Supplement: Supplementary file 6 — Additional file 6: Figure S6 CR mice cured by the combined therapy develop immune memory against tumor antigens expressed in the MH-22A tumor. There were totally eight tumor-bearing mice exhibiting CR after the combined therapy with APG-115 plus anti-PD-1 antibody (Fig. 4a). To assess immune memory, these animals were re-challenged by inoculating murine MH-22A liver tumor cells 3 weeks post the last treatment as detailed in the Materials and Methods section. Naïve C3H mice were inoculated with the tumor cells as the control. The tumor growth curves of the pooled (A) and individual mice (B and C) were presented. [file 40425_2019_750_MOESM6_ESM.docx]

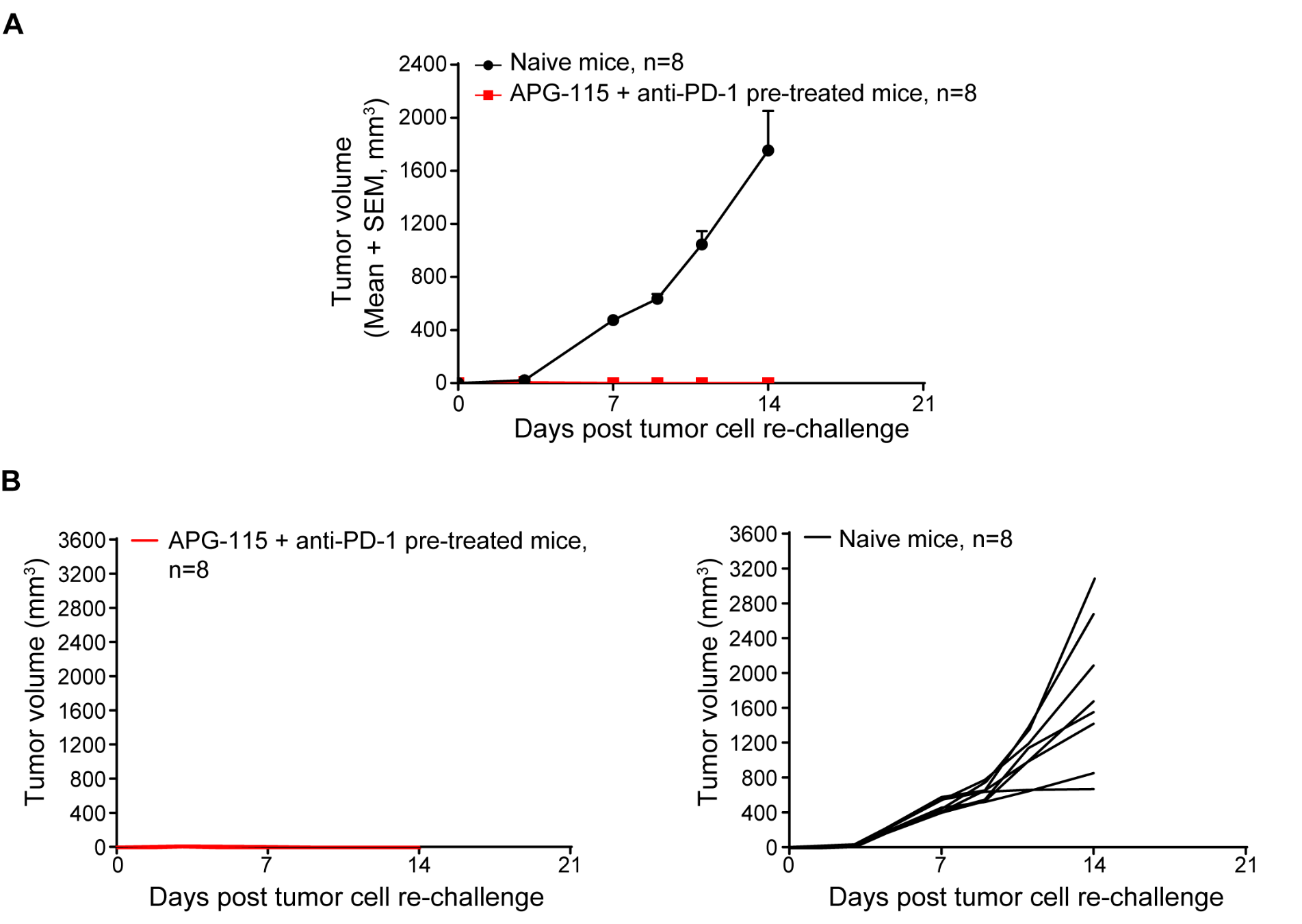


**Figure S6. CR mice cured by the combined therapy develop immune memory against tumor antigens expressed in the MH-22A tumor.** There were totally eight tumor-bearing mice exhibiting CR after the combined therapy with APG-115 plus anti-PD-1 antibody (Figure 4A). To assess immune memory, these animals were re-challenged by inoculating murine MH-22A liver tumor cells three weeks post the last treatment as detailed in the Materials and Methods section. Naïve C3H mice were inoculated with the tumor cells as the control. The tumor growth curves of the pooled (A) and individual mice (B and C) were presented.
