# Supplementary material for: MDM2 inhibitor APG-115 synergizes with PD-1 blockade through enhancing antitumor immunity in the tumor microenvironment
Source: J Immunother Cancer. 2019 Nov 28;7:327. doi: 10.1186/s40425-019-0750-6 (PMC6883539; doi:10.1186/s40425-019-0750-6)
Supplement: Supplementary file 7 — Additional file 7: Figure S7 Flow cytometry analysis of CD4+ T cells, NK cells, MDSC and Treg cells in the TME of syngeneic tumors with wild-type (A, MH-22A) or mutant (B, MC38) Trp53. Besides the frequency of TILs shown in Fig. 5, additional ones were shown here (n = 5 or 10). I + V indicates isotype control and vehicle of APG-115. [file 40425_2019_750_MOESM7_ESM.docx]

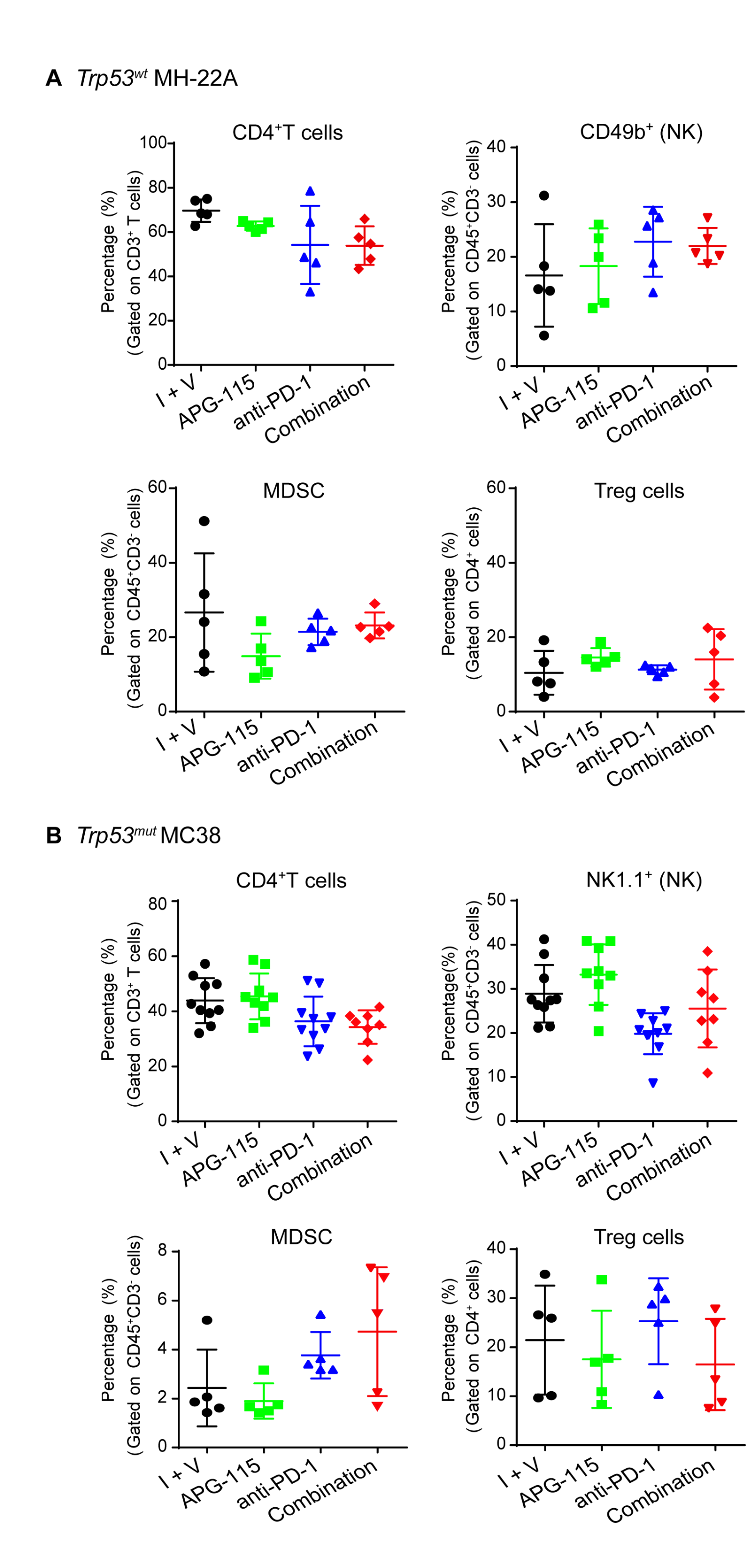


**Figure S7. Flow cytometry analysis of CD4^+^ T cells, NK cells, MDSC and Treg cells in the TME of syngeneic tumors with wild-type (A, MH-22A) or mutant (B, MC38) *Trp53*.** Besides the frequency of TILs shown in Figure 5, additional ones were shown here (*n* = 5 or 10). I + V indicates isotype control and vehicle of APG-115.
