# Supplementary material for: MDM2 inhibitor APG-115 synergizes with PD-1 blockade through enhancing antitumor immunity in the tumor microenvironment
Source: J Immunother Cancer. 2019 Nov 28;7:327. doi: 10.1186/s40425-019-0750-6 (PMC6883539; doi:10.1186/s40425-019-0750-6)
Supplement: Supplementary file 8 — Additional file 8: Figure S8 Combined treatment with APG-115 and anti-PD-1 increases tumor infiltrated CD4+ IFN-γ+ T cells. Mice with established MH-22A tumors were treated with APG-115 and anti-PD-1 as described in the legend of Fig. 4. Tumors were isolated on day seven after the first treatment and the expression levels of IFN-γ, TNF-α in T cells were analyzed by flow cytometry (n = 10/group). Shown are percentages of IFN-γ+, TNF-α+ within CD4+ T cells (A), and within CD8+ T cells (B). *P < 0.05 and ****P < 0.0001, by one-way ANOVA followed with Turkey’s multiple comparisons test. I + V indicates isotype control and vehicle of APG-115. [file 40425_2019_750_MOESM8_ESM.docx]

**
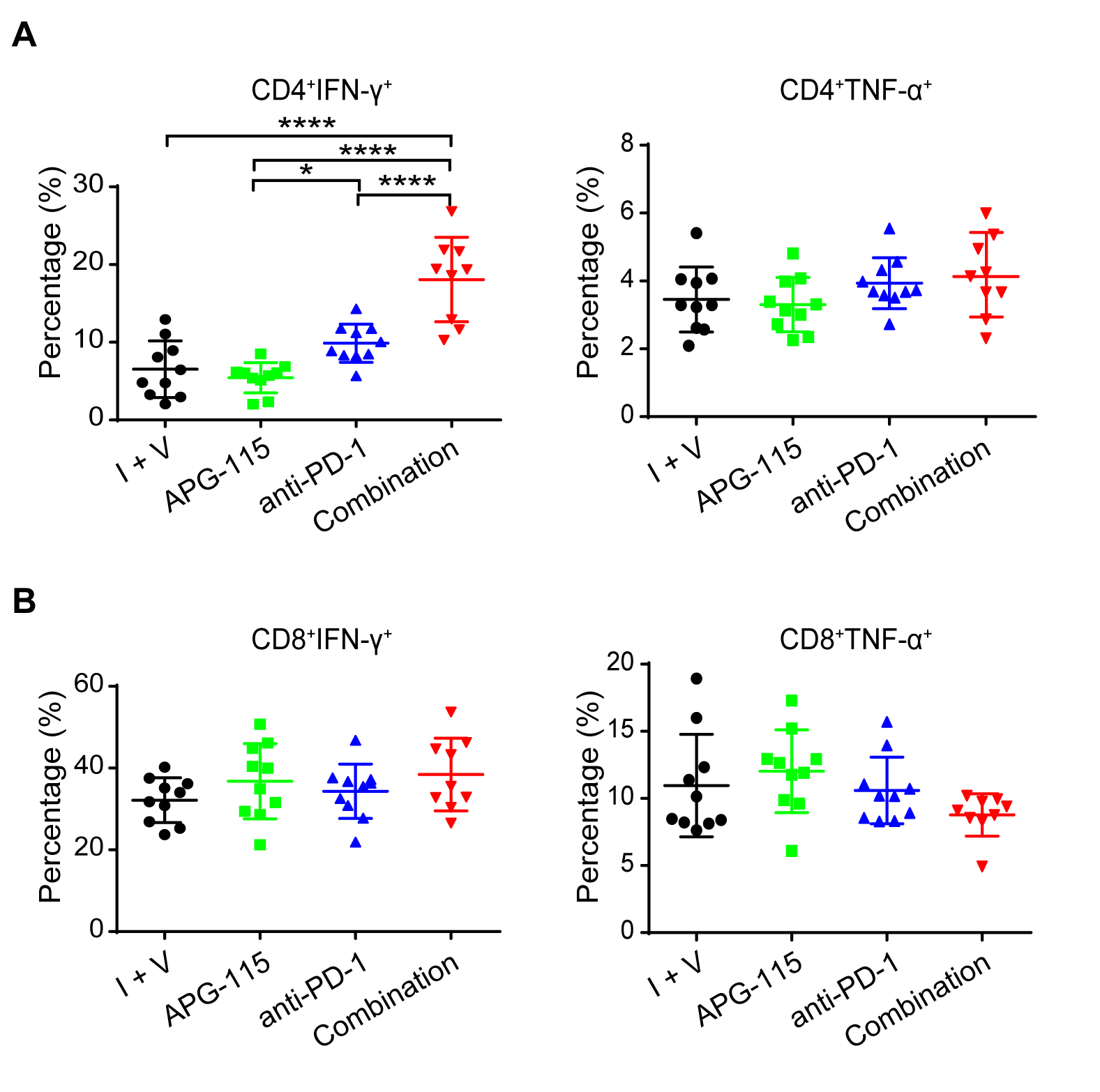
**

**Figure S8. Combined treatment with APG-115 and anti-PD-1 increases tumor infiltrated CD4^+^ IFN-γ^+^ T cells**. **.** Mice with established MH-22A tumors were treated with APG-115 and anti-PD-1 as described in the legend of Figure 4. Tumors were isolated on day seven after the first treatment and the expression levels of IFN-γ, TNF-α in T cells were analyzed by flow cytometry (n=10/group). Shown are percentages of IFN-γ^+^, TNF-α^+^ within CD4^+^ T cells (A), and within CD8^+^ T cells (B). *P < 0.05 and ****P < 0.0001, by one-way ANOVA followed with Turkey’s multiple comparisons test. I + V indicates isotype control and vehicle of APG-115.
